# Core–Shell Semiconductor-Graphene Nanoarchitectures for Efficient Photocatalysis: State of the Art and Perspectives

**DOI:** 10.1007/s40820-024-01503-4

**Published:** 2024-09-09

**Authors:** Jinshen Lan, Shanzhi Qu, Xiaofang Ye, Yifan Zheng, Mengwei Ma, Shengshi Guo, Shengli Huang, Shuping Li, Junyong Kang

**Affiliations:** Engineering Research Center of Micro-Nano Optoelectronic Materials and Devices, Ministry of Education, Fujian Key Laboratory of Semiconductor Materials and Applications, CI Center for OSED, Department of Physics, Xiamen University, Xiamen, 361005 People’s Republic of China

**Keywords:** Core–shell semiconductor-graphene, Nanoarchitecture, Photocatalysis, Driving force, Interface

## Abstract

Semiconductor photocatalysis holds great promise for renewable energy generation and environment remediation, but generally suffers from the serious drawbacks on light absorption, charge generation and transport, and structural stability that limit the performance. The core–shell semiconductor-graphene (CSSG) nanoarchitectures may address these issues due to their unique structures with exceptional physical and chemical properties. This review explores recent advances of the CSSG nanoarchitectures in the photocatalytic performance. It starts with the classification of the CSSG nanoarchitectures by the dimensionality. Then, the construction methods under internal and external driving forces were introduced and compared with each other. Afterward, the physicochemical properties and photocatalytic applications of these nanoarchitectures were discussed, with a focus on their role in photocatalysis. It ends with a summary and some perspectives on future development of the CSSG nanoarchitectures toward highly efficient photocatalysts with extensive application. By harnessing the synergistic capabilities of the CSSG architectures, we aim to address pressing environmental and energy challenges and drive scientific progress in these fields.

## Highlights

- The constructions under internal and external driving forces were introduced and compared with each other.
- The physicochemical properties were analyzed for the assessment of crystalline quality and photoelectric characteristics.
- The photocatalytic applications, mechanisms, and developments of the core-shell semiconductor-graphene nanoarchitectures were illustrated in detail.

## Graphical abstract

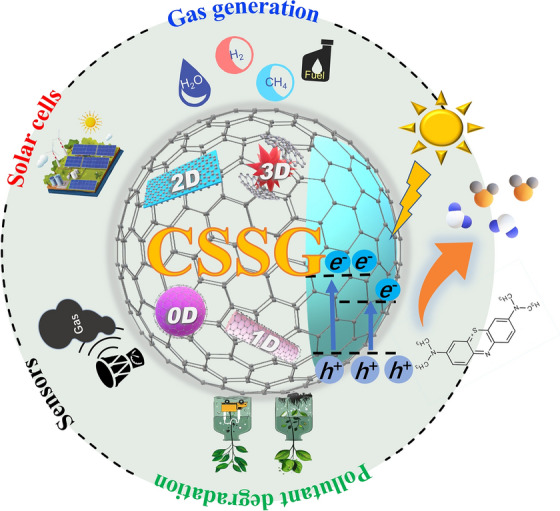

## Introduction

The environmental and energy challenges have become critical global issues for the rapid development of economy and social activity in the twenty-first century. The semiconductor photocatalysts emerge as a solution as they can directly convert solar energy into chemical energy as well as catalytically degrade organic pollutants into harmless substances and renewable fuels [1–3]. However, single semiconductor materials often suffer from poor solar absorption, low generation rate of the electron–hole pairs, fast charge recombination and structural instability that restrict their performances. To overcome these shortcomings and unlock the full potential of semiconductors, heterogeneous engineering, elemental doping and surface modification have been widely adopted [4–12]. In particular, the construction of core–shell nanoarchitectures has gained prominence due to its large heterojunction area with exceptional chemical and physical properties, which allows for the enhancement and modification of material properties through the synergy of the different components [13–16].

Graphene, as a unique hexagonal *sp*^2^ hybrid carbon network, has an extraordinary surface area of 2630 m^2^ g^−1^, a high charge carrier mobility of 2 × 10^5^ cm^2^ V^−1^ S^−1^, an exceptional thermal conductivity of 5000 W m^−1^ K^−1^, a good optical transparency efficiency of 97.7%, superior mechanical properties with a large Young’s modulus exceeding 1.0 TPa, and strong chemical stability [17–21]. These unique characteristics make graphene and its derivatives (graphene oxide and reduced graphene oxide) an indispensable choice in the semiconductor modification, sparking significant interest in the field of solar cell [22, 23], lithium-ion battery [24–27], supercapacitor [28, 29], photodetector [30, 31], and photocatalysis [32–35]. In contrast to other structures, the core–shell semiconductor-graphene (CSSG) nanoarchitectures own a large junction area with a built-in electric field, which promotes electron–hole pair separation in the semiconductor component and directs charge migration from semiconductor to the conductive graphene. Secondly, the CSSG nanoarchitectures can intensify or introduce new chemical or physical capabilities not present in the individual core and shell materials, such as the formation of C-metal bonds that change the optoelectronic properties of the semiconductor component [36, 37]. Thirdly, the shell can act as a protective layer that maintains the structural integrity and properties of the core by excluding environmental influence, limiting volume expansion and preventing aggregation into large particles. Fourthly, the CSSG nanoarchitectures can significantly improve light absorption and utilization for the transparent characteristic of the graphene sheets. Last but not the least, the CSSG nanoarchitectures can selectively percolate ions or molecules onto the core, allowing for controlled interactions. Thanks to these advantages and the rapid development of construction methods and characterization techniques, the CSSG nanomaterials have been synthesized in various morphologies and gained considerable achievements.

Although tremendous efforts have been devoted to the fabrication and application of CSSG nanomaterials, much research interest was focused on a specific sample [36–40]. The related review is mainly devoted to the energy conversion and storage in lithium-ion batteries and supercapacitors [41–44], while that on photocatalysis is rarely reported. Given that the research on these hybrid materials is progressing fast, a review about the selection of appropriate fabrication methods to obtain desired CSSG nanoarchitectures based on the photocatalytic application is greatly needed. The suitability of photocatalytic system and the mechanism illustration should be carried out to promote the implementation of this technology. This review highlights the CSSG nanoarchitectures for photocatalytic performance. Specifically, the section following introduction, i.e., Sect. 2, classifies the CSSG nanoarchitectures by the dimensionality. In Sect. 3, the construction methods of the CSSG nanoarchitectures are introduced and compared with each other. In Sect. 4, the physicochemical properties of the CSSG nanoarchitectures are analyzed, with a focus on the binding effect, the amount and lattice characteristics of the graphene sheets, the photoelectric modulation of the semiconductor component, the defect states and charge transport of the hybrid materials. In Sect. 5, the photocatalytic applications of the CSSG nanoarchitectures, including the degradation of organic pollutants, the generation of hydrogen gas, and the reduction of carbon dioxide are discussed, where the photocatalytic mechanisms are illustrated in order to reveal the enhancement. In Sect. 6, a summary and future developments of the CSSG nanoarchitectures on photocatalysis are provided.

## Classification of CSSG Nanoarchitectures

**Scheme 1:**
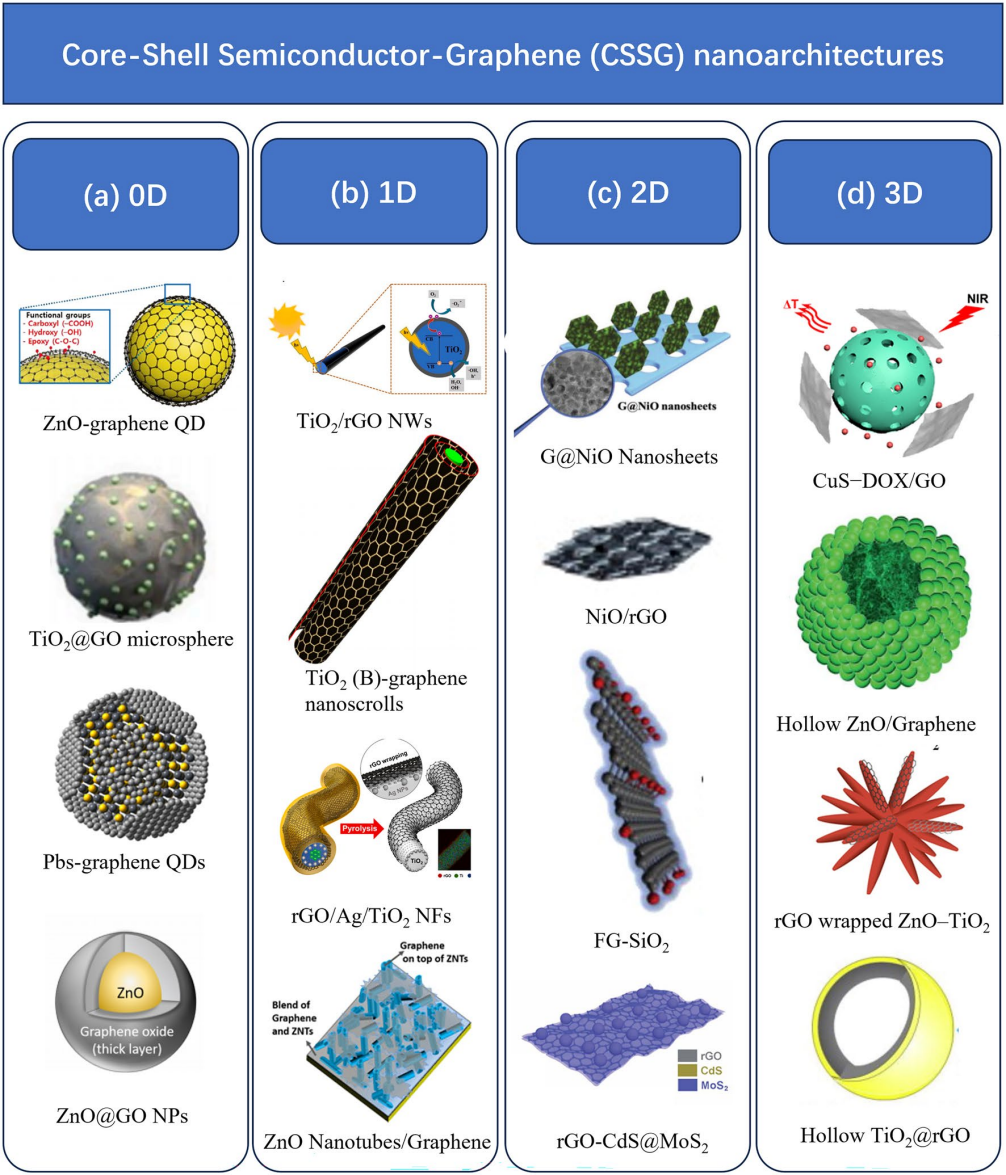
CSSG nanomaterials in 0D, 1D, 2D, and 3D architectures. **a** 0D nanostructure of ZnO-Graphene quantum dots (reproduced with permission from Ref. [45]. Copyright 2021 Elsevier Publishing), TiO_2_@GO microsphere (reproduced with permission from Ref. [46]. Copyright 2022 Elsevier Publishing), PbS/G QDs (reproduced with permission from Ref. [47]. Copyright 2015 ACS Publishing), ZnO@GO NPs (reproduced with permission from Ref. [48]. Copyright 2020 Elsevier Publishing); **b** 1D nanostructure of TiO_2_/rGO NWs (reproduced with permission from Ref. [50]. Copyright 2022 Elsevier Publishing), TiO_2_ (B)-graphene nanoscrolls (reproduced with permission from Ref. [51]. Copyright 2014 Elsevier Publishing), rGO/Ag/TiO_2_ NFs (reproduced with permission from Ref. [52]. Copyright 2022 Elsevier Publishing), ZnO Nanotubes/Graphene (reproduced with permission from Ref. [53]. Copyright 2017 ACS Publishing); **c** 2D nanostructure of G@NiO Nanosheets (reproduced with permission from Ref. [60]. Copyright 2017 WILEY–VCH Publishing), NiO/rGO (reproduced with permission from Ref. [61]. Copyright 2019 Elsevier Publishing), FG-SiO_2_ (reproduced with permission from Ref. [62]. Copyright 2014 Elsevier Publishing), rGO-CdS@MoS_2_ (reproduced with permission from Ref. [63]. Copyright 2021 RSC Publishing); **d** 3D nanostructure of CuS−DOX/GO (reproduced with permission from Ref. [64]. Copyright 2017 ACS Publishing), Hollow ZnO/Graphene (reproduced with permission from Ref. [65]. Copyright 2016 RSC Publishing), rGO wrapped ZnO–TiO_2_ (reproduced with permission from Ref. [66]. Copyright 2021 MDPI Publishing), Hollow TiO_2_@rGO (reproduced with permission from Ref. [67]. Copyright 2015 Elsevier Publishing)

The CSSG nanoarchitectures can be divided based on the dimensionality, including 0-dimension (0D), 1-dimension (1D), 2-dimension (2D) and 3-dimension (3D), as presented in Scheme 1. The CSSG nanoarchitecture in 0D contains the morphologies of particle, sphere, quantum dot (QD), quasi-sphere and quasi-particle [36, 38, 45–49], while that in 1D presents in the forms of wire, rod, fiber, and tube [38, 40, 50–59], and that in 2D dominates in the morphologies of thin film and nanosheet [60–63]. The CSSG nanoarchitecture in 3D appears in diverse morphologies, such as rose, walnut, urchin, and hollow sphere [37, 64–70]. However, the 3D architecture is generally constructed by the 0D, 1D, or 2D materials. For example, the flower-like configuration of c-Ga_2_O_3_@rGO core–shell nanostructures is composed of 2D nanosheets [68]. In the CSSG nanoarchitectures, the semiconductor component may be in the core or in the shell, or the graphene sheet inserts among the semiconductor component. Except for the semiconductor and graphene, other chemical components may be also included for the enhanced performance, such as noble metal nanoparticles (NPs) [52, 71]. The graphene can be single- or multilayered graphene nanosheets, graphene oxide or reduced graphene oxide. For simplification, in the following discussion, the graphene refers to the graphene and its derivatives, while the G, GO, and rGO refer to the specific graphene, graphene oxide, and reduced graphene oxide.

For the photocatalytic application, the 0D CSSG catalysts own a large specific surface area and a stable structure, which are beneficial for the reactant absorption and recyclability. Nevertheless, the catalysts are generally dispersed in the solution, thus are hard to be recovered, and may induce secondary pollution. The 1D and 2D CSSG catalysts may be designed as an array standing on the substrates [40, 60]. This can be reused conveniently just by immersing the substrates in the solution and picking up, but the array uniformity may be limited by the construction method. The 3D CSSG catalysts can expand orientations with large interface area, but the structural stability and accessible surface-active sites need to be considered. The designed components and structures should be based on the material function, recyclability, and viable synthesis methods.

## Construction Methods

The CSSG nanomaterials have been constructed by a lot of methods that are driven by the internal force or external force. For example, most of the CSSG nanomaterials are synthesized by the electrostatic self-assembly, which is due to the internal force of electrostatic interparticle attraction [21, 69, 72–77]. On the other hand, the CSSG nanomaterials with perfect crystalline characteristics are widely fabricated by chemical vapor deposition (CVD) that can be considered as the external force for the high-temperature and gaseous environment [20, 36, 60, 78, 79]. Nevertheless, the both kinds of driving force may coexist in the synthesis of a specific sample. In this section, the widely adopted construction methods of the CSSG nanomaterials will be introduced, and the advantages and disadvantages of each method will be disclosed in detail.

### Electrostatic Self-Assembly

**Fig. 1:**
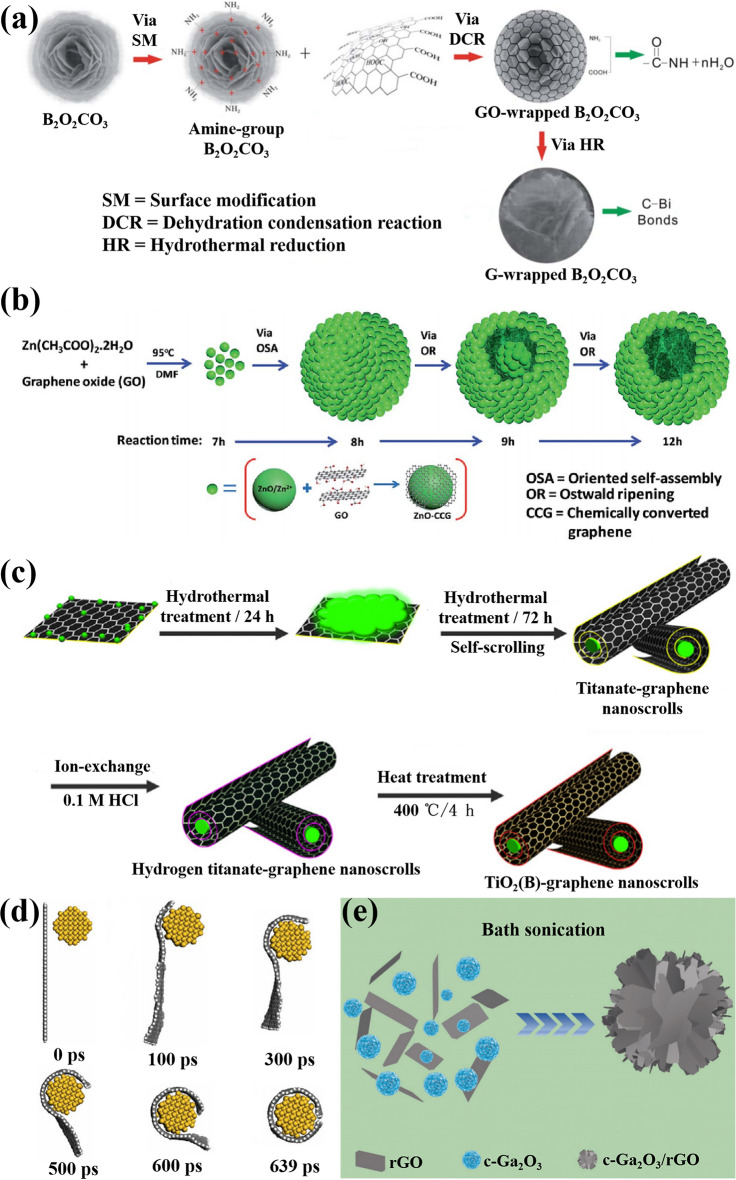
Construction methods of the CSSG nanoarchitectures by the internal force: **a** Electrostatic self-assembly (reproduced with permission from Ref. [37]. Copyright 2014 RSC Publishing). **b** Oriented self-assembly and Ostwald ripening (reproduced with permission from Ref. [65]. Copyright 2016 RSC Publishing). **c** Hydrothermal treatment and self-scrolling (reproduced with permission from Ref. [51]. Copyright 2014 Elsevier Publishing). **d** Van der Waals interaction (reproduced with permission from Ref. [59]. Copyright 2010 Elsevier Publishing). **e** Bath sonication (reproduced with permission from Ref. [68]. Copyright 2023 Wiley Online Library Publishing)

The electrostatic self-assembly is a widely adopted method in the synthesis of CSSG nanomaterials. It may consist of three steps, as shown in Fig. 1a [37]. Firstly, the semiconductor surface is modified with a positive potential. Secondly, a GO layer coats onto the semiconductor by the electrostatic attraction between the positive potential of semiconductor and the intrinsic negative potential of GO [80]. Lastly, the GO shell is converted to graphene. In the first step, the poly(allylamine hydrochloride) (PAH) solution or amino-propyl-trimethoxysilane solution (APTES) can be used as the modifier to induce the cationic surface of the semiconductor [38, 40, 72, 81], while in the last step, the reduction can be performed by annealing at high temperatures or by reduction agents, such as hydrazine [40, 54]. As the electrostatic attraction plays a critical role in the core–shell coupling, this method can be considered as driven by the internal force. The whole process can be carried out in solution, which is convenient for the operation, for different components and morphologies, and for a large scale. Nevertheless, it is difficult to control interfacial growth, and the residuals of the molecular linkers may counteract the performance. In order to reduce or exclude the negative effects of molecular linkers, further action can be taken on the achieved samples, such as thermolysis, acid modulation, and isotope substitution [57, 82, 83].

### Oriented Self-Assembly and Ostwald Ripening

The process of oriented self-assembly and Ostwald ripening is a hydrothermal method that can produce CSSG nanomaterials with various morphologies in solid and hollow microspheres (MSs). As displayed in Fig. 1b [65], the core–shell ZnO/graphene NPs are achieved by the covalent reaction of zinc acetate dihydrate and GO in dimethyl formamide (DMF) medium. The NPs start to aggregate preferentially and self-assemble into metastable MSs by oriented attachment to minimize the total surface energy. On prolonging the reaction time, the solid MSs convert into core–shell structured hollow MSs and finally, to hollow MSs by the loss of smaller NPs into larger NPs via Ostwald ripening process. In this method, the formation of the CSSG nanomaterials is merely driven by the covalent reaction as the internal force, and the various morphology can be achieved in the same solution by just optimizing the reaction time and temperature, which is more convenient than that for electrostatic self-assembly. However, the covalent reaction at the low temperature is only possible for some active specific reactants, which limits the material choice. Moreover, the oriented self-assembly and Ostwald ripening induce difficulty in structure control of the final product. The external force, such as an electric or magnetic field, can be applied to direct the growth orientation and increase the crystal quality [84].

### Hydrothermal Treatment and Ion-Exchange/Self-Scrolling/Self-Stocking/Annealing

The method uses the amphiphilic and self-scrolling nature of the GO nanosheets as the internal force [59, 85]. As illustrated in Fig. 1c [51], the titania NPs are likely to anchor on the GO nanosheets to form homogeneous suspension in alkali solution under ultrasonication. After hydrothermal treatment, the amorphous titanate intermediates successively contract and transform to titanate nanowires (NWs), while the graphene nanosheets are scrolled up to encapsulate titanate NWs inside. After ion-exchange and annealing, the core–shell titanate/graphene NWs are synthesized. The final annealing can be performed in solution [86–90], air [51, 91], vacuum [92], or special gas atmosphere [80], which plays an important role in improving the crystal quality and interfacial affinity between the core and the shell. As it is a one-step hydrothermal method, the operation is simpler than the others, and the product can be acquired in any amount and morphology. Nevertheless, the reaction should take place at a high temperature, generally close to the temperature capability of an autoclave. In addition, the reactants may act as the residue in the products, and the final morphology is also uncontrollable as it is a spontaneous process.

### Van Der Waals Interaction

The method limits to the adjacent small objects. As shown in Fig. 1d [59], once the diameter of the NWs (yellow dots) reaches a threshold (10 nm), the surface adsorption stress of the NWs, which comes from van der Waals force as an internal driver, is introduced to bend graphene nanosheets to roll up and cover on the NW surface. This construction takes place in solution and can be modulated by the solute concentration and temperature [58], which is convenient for the operation. However, the shell thickness is difficult to be modulated, and the interface may be loose for the weak binding. Surface modification may be applied to improve the interaction and binding force between the core and the shell.

### Bath Sonication

The CSSG nanomaterials grown by bath sonication generally use the natural opposite zeta potentials of reactants as the internal force for the core and shell. As shown in Fig. 1e [68], the zeta potentials of c-Ga_2_O_3_ and rGO are + 48.6 and − 58.3 mV, respectively. When the two components are dispersed in the solution by the sonication, they will assemble into a flower-like configuration of c-Ga_2_O_3_/rGO core–shell nanostructures for the electrostatic attraction. The experiment is usually performed in the solution at room temperature [68, 93, 94], which is much easier than others. However, the potential difference of the components should be as large as possible, so that they can exclude interference and combine together with an intimate interface. Moreover, the shell thickness is difficult to be increased as the electrostatic force will be reduced for the long distance.

### Chemical Vapor Deposition

**Fig. 2:**
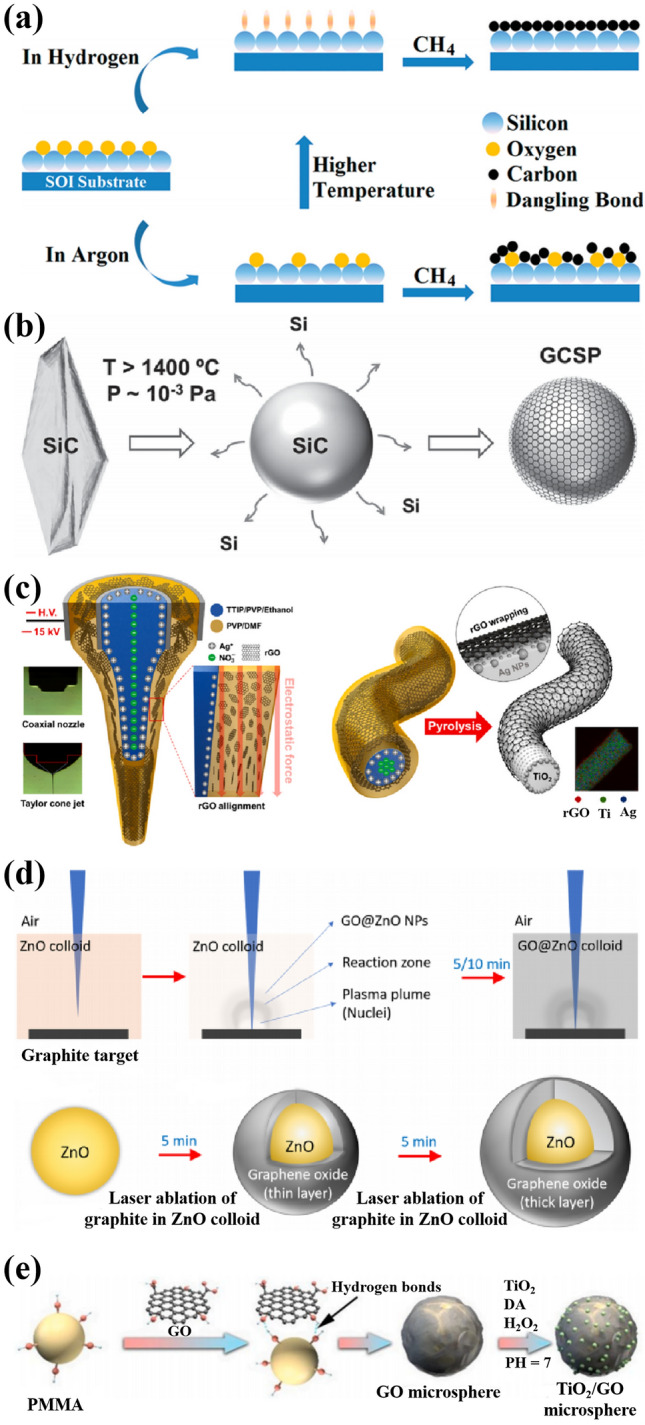
Construction methods of the CSSG nanoarchitectures by the external or internal force: **a** CVD (reproduced with permission from Ref. [95]. Copyright 2012 AIP Publishing). **b** Pyrolysis (reproduced with permission from Ref. [96]. Copyright 2015 WILEY–VCH Publishing). **c** Electrospinning and annealing (reproduced with permission from Ref. [52]. Copyright 2022 Elsevier Publishing). **d** Laser ablation (reproduced with permission from Ref. [48]. Copyright 2020 Elsevier Publishing). **e** Template method (reproduced with permission from Ref. [46]. Copyright 2022 Elsevier Publishing)

The chemical vapor deposition (CVD) is widely adopted to grow CSSG nanomaterials in different component with different morphology in a high quality. In a general process, the semiconductor compound is loaded in a quartz tube. Then, the tube is heated to a reaction temperature. Afterward, the hydrogen and argon gases are introduced to the reactor for some time in order to clean and activate the semiconductor surface. Finally, the gas-phase carbon source (methane, ethylene, etc.) is also introduced to the reactor for the graphene growth. Therefore, the CVD is mainly driven by the external force for the high-temperature and gaseous environment. The thickness of the graphene shell can be modulated by the reaction time, temperature and gas flux. It is conjectured that the semiconductor surface will produce dangling bonds in the gas atmosphere at high temperatures, which will stick strongly to the C atoms from the decomposed hydrocarbons. The surface-adsorbed C atoms accumulate to form graphene. The hydrogen gas is not indispensable for the reaction, but it can reduce the reaction temperature, produce more active sites on the semiconductor surface, and bring the formation of large-size graphene, as shown in Fig. 2a [95]. Because the gas sources tend to react with the oxygen in air, the experiment can only be performed in the sealed tubes.

### Pyrolysis

The CSSG nanomaterials achieved from pyrolysis requires that the core semiconductor contains the carbon component, and the surface layer of the semiconductor inclines to decompose at high temperatures [19, 96]. As displayed in Fig. 2b [96], the surface of SiC particles decomposes into silicon and carbon atoms at high temperatures in argon atmosphere, leading to the escape of silicon atoms and the reconstruction of C ions to from a graphene layer on the particles. In the experiment, the temperature, acting as the external driving force, should be high enough to decompose the compound, and the performance needs to be in vacuum or inert gas to avoid the oxygen reaction.

### Electrospinning and Annealing

Using a core–shell nozzle, the CSSG NWs or nanofibers (NFs) can be achieved by the electrospinning method [52, 56]. As displayed in Fig. 2c [52], a negative electric potential (− 15 kV) is placed between the nozzle and a collector. A polyvinylpyrrolidone (PVP)/rGO solution is ejected through the outer part of the nozzle and a AgNO_3_/TTIP(Titanium isopropoxide)/PVP composite solution through the inner part. The electrohydrodynamic alignment of rGO and transportation of Ag^+^ occur during the electrospinning process, constructing core–shell NFs. Subsequent thermal treatment induces interfacial assembly of the rGO flakes on the surface of Ag/TiO_2_ NFs. By turning the components, the method is convenient to construct different CSSG NFs and NWs, even the graphene in the core. Moreover, as driven by the external force, the electrospinning process can be replaced by other strategy, such as microfluidic-spinning [57]. However, in order to obtain a steady structure out of the nozzle, the sheath and core fluids are usually mixed with polymers, such as PVP. This may introduce impurity and deteriorate the performance of the final products. The last thermal treatment seems more important to remove the polymers.

### Laser Ablation

Laser ablation of graphite target immersed in semiconductor colloid/deionized water medium can lead to the formation of GO-encapsulated semiconductor NPs, which follows the thermal evaporation mechanism. As shown in Fig. 2d [48], laser ablation on graphite plate exfoliates carbon layers to produce plasma plume rich in C ions. The plasma-induced water vapor and ionized ZnO NPs from ZnO colloid interact with the ablated carbon ions, resulting in the assembly of the latter carbon species on the surface of ZnO NPs and formation of core–shell ZnO/GO. The process is mainly driven by the laser exfoliation as the external force. By changing the colloid components and extending carbon ablation, the heterogeneous materials and shell thickness can be changed, which is convenient for the modulation. However, the colloids should be small enough in order to be wrapped uniformly.

### Template Method

The CSSG nanomaterials can also be achieved by depositing the components on a template. As shown in Fig. 2e [46], by using the poly(methyl methacrylate) (PMMA) sphere as a hard template, GO nanosheets spontaneously wrap the surface of PMMA spheres, as driven by the hydrogen bonding and van der Waals force as the internal force. On this basis, the TiO_2_ NPs are directly immobilized onto GO MS surface by using the dopamine (DA) as bridge, thus obtaining the 3D TiO_2_@GO core–shell sphere. This method is convenient to adjust the CSSG morphology by using the pre-designed template structure, and the solid cores can be turned into void by annealing or etching. However, the binder is introduced for the combination, which may decline the physicochemical properties of the CSSG materials. Further action, such as thermolysis and acid modulation, can be performed to remove the binders, as that in the electrostatic self-assembly.

Besides the methods described above, the CSSG nanomaterials can also be synthesized by other ways, such as sol–gel synthesis [97, 98], ball-milling [99], spray drying [100–102], microwave-assisted synthesis [103–105], and so on [106–108]. For comparison, the diverse methods as well as the advantages and disadvantages of each one are listed in Table 1. It is believed that more and more methods will be adopted, which will enrich the construction, diversify the pattern, and expand the application of the CSSG nanomaterials.

**Table 1:** The comparison of synthesis methods of CSSG materials

Synthesis methods	Advantages	Disadvantages	References
Electrostatic self-assembly	Solution processable, large scale	Residuals of the molecular linkers, difficult to control interfacial growth process	[21, 37, 38, 40, 72–77]
Hydrothermal treatment and ion-exchange /self-scrolling /self-stocking /annealing	Solution processable, large scale	Special reactants, residuals of the reactants, uncontrollable structure	[50, 55, 59, 80, 86–92]
Covalent reaction	Solution processable, large scale	Residuals of the molecular linkers	[106]
Oriented self-assembly and Ostwald ripening	Solution processable, large scale	Special reactants, uncontrollable structure	[65]
Van der Waals interaction	Solution processable, large scale	Limited shell thickness, weak binding at the interface	[59, 60]
Bath sonication	Solution processable, large scale	Special reactants, limited shell thickness	[93]
In situ reduction and dealloying	Solution processable, large scale	Residuals of the reactants, un-uniformity	[70]
Sol–gel synthesis	Solution processable, large scale	Residuals of the reactants	[97]
Chemical vapor deposition	Good crystallization, tunable dimension, immobilization	Energy-intensive, special circumstance	[20, 36, 60, 95]
Pyrolysis	Thin shell layer	Energy-intensive, special sample	[19, 96]
Spray drying	Easy operation, low cost	Mixed phases	[100–102]
Electrospinning and annealing	Easy operation, low cost	Mixed phases	[52, 56]
Microfluidic spinning and annealing	Easy operation, low cost	Mixed phases	[57]
Laser ablation	Good crystallization, tunable dimension	Energy-intensive, limited core size	[48]
Template	Tunable structure	Residuals of the binders	[46]
Microwave-assisted synthesis	Uniform distribution of energy inside the reaction vessel, energy saving, high reproducibility, and excellent control over experimental parameters	Residuals of the reactants,	[103–105]
Adsorption–deposition	Solution processable, large scale	Residuals of the binders, limited thickness	[107]
Precipitation	Solution processable, large scale	Residuals of the reactants, morphology uncontrollable	[108]
Ball-milling	Easy operation, large scale	Special reactants, residuals of the reactants	[99]

## Physicochemical Properties

In comparison with the pristine semiconductors, the control of the CSSG nanoarchitectures can endow them with large surface area, abundant surface states, extended light harvesting, improved heat and electronic conduction, and even vectoring charge separation and migration, all of which are beneficial for their optoelectronic and photocatalytic performances. In this section, the structure characteristics of the CSSG nanomaterials, the method to test the layer number and interlayer spacing of the graphene nanosheets, the modulation of optical and electrochemical properties, and the assessment of bandgap, defect states, charge transport, and recombination rate will be provided by comparison and analysis of the morphology and structure, optical, and electrochemical properties of the CSSG nanoarchitectures.

### Morphology and Structure

**Fig. 3:**
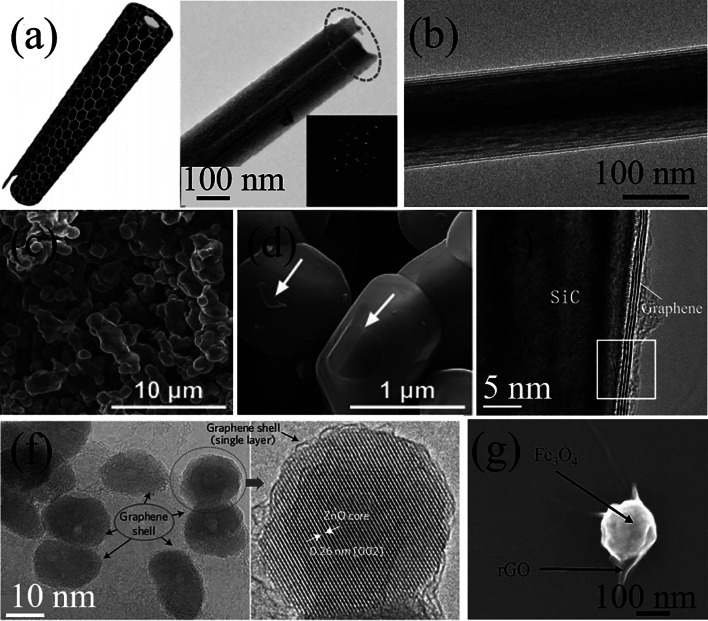
Morphology and structure of the CSSG materials: **a** Structural illustration and TEM image of TiO_2_ (B)-graphene nanoscrolls (the inset shows the corresponding SAED pattern) (reproduced with permission from Ref. [51]. Copyright 2014 Elsevier Publishing). **b** TEM image of a TiO_2_/rGO NW. **c** SEM image of SiC/G NPs. **d** Magnified SEM image of SiC/G NPs (the arrows mark the peeled graphene or the graphene wrinkle). **e** HRTEM image of a SiC/G NP (reproduced with permission from Ref. [19]. Copyright 2014 RSC Publishing). **f** HRTEM image of ZnO/G QDs (reproduced with permission from Ref. [49]. Copyright 2012 Macmillan Publishers Limited Publishing). **g** SEM image of a Fe_3_O_4_/rGO NP (reproduced with permission from Ref. [109]. Copyright 2023 Elsevier Publishing)

The morphologies of CSSG materials are closely related to the synthetic method. For example, by hydrothermal treatment and ion-exchange, graphene nanosheets can be scrolled up to encapsulate titanate NWs inside, resulting in scrolled architectures. The heat treatment at low temperature (400 °C) induces a porous structure with a visible gap between the core and the shell (Fig. 3a) [51], while a tightly binding architecture is achieved at a high temperature (700 °C) (Fig. 3b) [50, 54]. Generally, by the construction methods at high temperatures, such as pyrolysis and CVD, the covered graphene layer is smooth and wraps the semiconductor core tightly with perfect contact interfaces, where the graphene monolayers can be clearly observed (Fig. 3c–e) [19]. However, for the hydrothermal growth and other construction methods at relatively low temperatures, the CSSG architectures usually display wrinkled and rough surface texture, though the layer number of the graphene can be also accurately controlled (Fig. 3f, g) [49, 109].

**Fig. 4:**
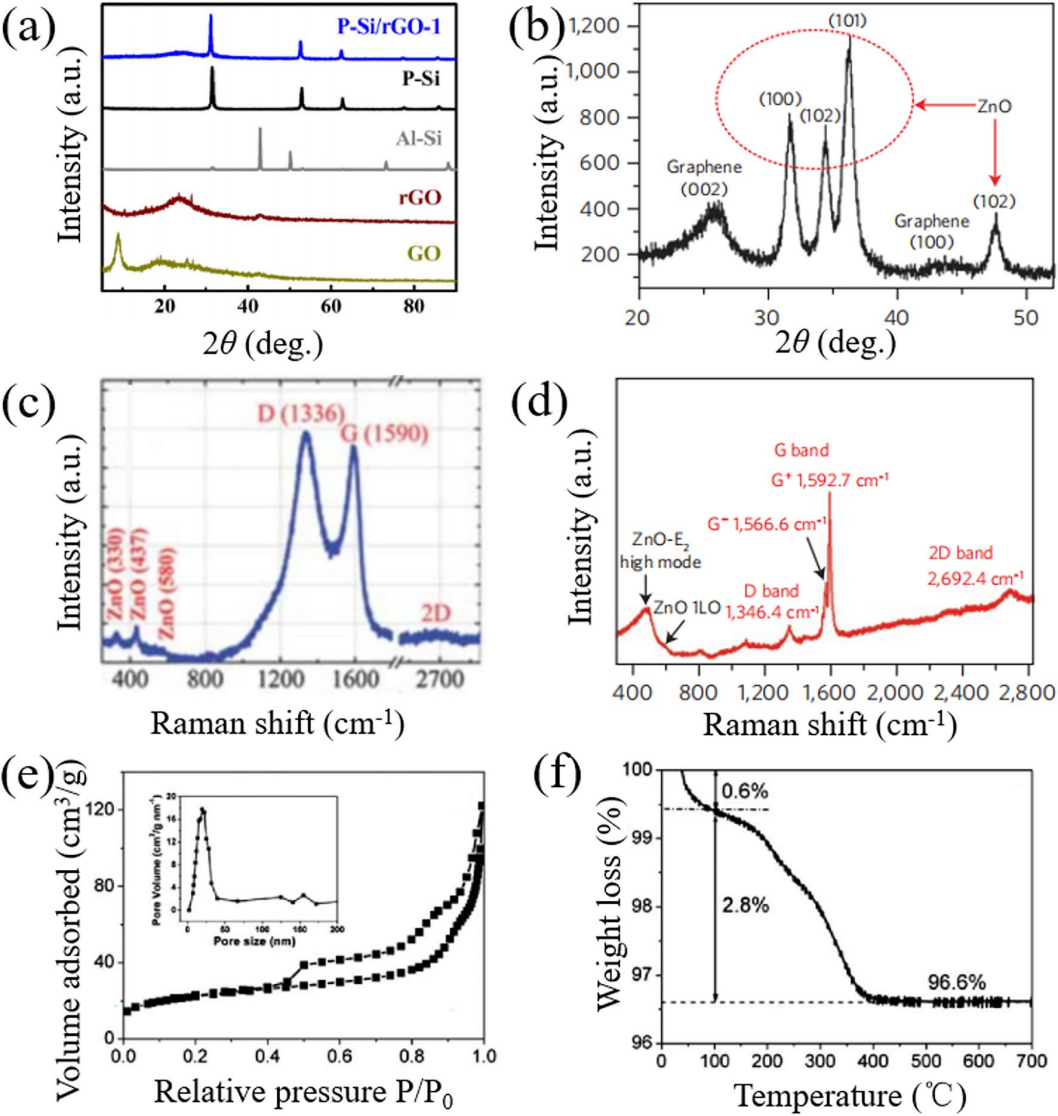
Structural analysis of the CSSG materials: **a** XRD patterns of GO, rGO, Al-Si powder, porous Si, and porous Si/rGO (reproduced with permission from Ref. [70]. Copyright 2017 Tsinghua University Press and Springer-Verlag Berlin Heidelberg Publishing). **b** XRD pattern of ZnO/G QDs (reproduced with permission from Ref. [49]. Copyright 2012 Macmillan Publishers Limited Publishing). **c** Raman spectra of ZnO/G NPs (reproduced with permission from Ref. [38]. Copyright 2013 RSC Publishing). **d** Raman spectra of ZnO/G QDs (reproduced with permission from Ref. [49]. Copyright 2012 Macmillan Publishers Limited Publishing). **e** Nitrogen isotherm adsorption–desorption curve of a-Fe_2_O_3_/G NPs. (The inset shows their pore size distributions.) **f** Thermogravimetric analysis of a-Fe_2_O_3_/G NPs (reproduced with permission from Ref. [40]. Copyright 2011 RSC Publishing)

Except for electron microscopy, the crystal quantity and graphene content of the CSSG nanomaterials can also be examined by XRD patterns, Raman, and infrared spectra. As observed in Fig. 4a [70], the XRD pattern of GO shows an intense and a relatively weak diffraction peaks in the 2*θ* of 9.5° (001) and 19.0° (002), which are due to the oxygenated functional groups and the amorphous nature of GO, while for G and rGO, the XRD pattern also presents two diffraction peaks at 22.0° and 41.0° that are corresponding to (002) and (001) plane of the nanosheets. The peak position and intensity are changed with the depletion of functional groups, the exfoliation of multiple layers and the variation of interlayer spacing. The diffraction peaks of the graphene shell are generally depressed by the semiconductor core for the small amount of the shell layer. But for the CSSG nanomaterials with high crystal quality, the XRD pattern generally contains the diffraction peaks of the core and the shell (Fig. 4b) [49]. The average crystallite size, lattice parameters, and interplanar spacing of the core and the shell can be estimated from the patterns [38, 48, 110]. Kulinich et al. found that the graphene layers formed atop ZnO cores can reduce the density of defects and disorders, decrease the anisotropic tensile stress, and improve the crystallinity of the ZnO cores [48]. However, these effects generally occur in the hard construction condition (laser ablation, pyrolysis, CVD, high-temperature annealing). In the mild conditions (electrostatic self-assembly, hydrothermal growth, etc.), the crystal features of the semiconductor cores are rarely influenced by the coating layers.

Raman scattering is commonly used to characterize carbon-based materials. Except for the scattering peaks of the semiconductor cores, Raman spectra of the CSSG nanomaterials contain characteristic bands of the graphene shells that are sensitive to the structural quality and layer number of the graphene as well as the interaction between the core and the shell. Figure 4c shows the Raman spectra of ZnO/G NPs [102], where the dominate D band (1336 cm^−1^) is corresponding to the non-*sp*^2^ carbon bonding such as atomic-scale defects or lattice disorder of graphene, while the G band (1590 cm^−1^) and 2D band (2675 cm^−1^) are the characteristics of *sp*^2^ hybridization of the C–C/C=C bonds in graphene. The intensity of G band and 2D band can be used to determine the number of graphene layers, whereas the intensity ratio of D band to G band (*I*_D_/*I*_G_) indicates disorder or local defects in the graphene layers that is widely applied in the assessment of the crystal quality. Chen et al. found a clear increase of *I*_D_/*I*_G_ from 0.95 for GO to 1.44 for G in hematite/G NPs, which is attributed to the formation of new and smaller *sp*^2^ domains during the reduction [91]. Zhang et al. got an *I*_D_/*I*_G_ ratio of 1.06 for commercial ZnO/G and 0.91 for hexagonal ZnO/G [72]. The difference suggests that the quality of graphene in hexagonal ZnO/G is higher than that in commercial ZnO/G and the structure of graphene in commercial ZnO/G is damaged in some degree after thermal annealing. The effect is also observed in TiO_2_/G from TiO_2_/GO by hydrothermal treatment [21]. Xu et al. found that the *I*_D_/*I*_G_ ratio of Cu_2_O/rGO is larger than that of GO nanosheets [90], demonstrating a decrease in the average size of the *sp*^2^ domains upon reduction of the exfoliated GO. Kim et al. noticed that the *I*_D_/*I*_G_ ratio of Al_2_O_3_/graphite decreased with increasing growth time, implying better multilayered graphene quality with the growth time [78]. Except for the intensity, the D and G bands will be shifted for the electronic interaction or electron transferring between the graphene shell and the semiconductor core. Moreover, both the double degenerate bands may divide into two sets of sub-bands (Fig. 4d) [49], D^+^ and D^−^ as well as G^+^ and G^−^, due to the strain induced symmetry breaking of the bending graphene layer on the semiconductor surface, with polarization along and perpendicular to the strain.

One of the important roles of the graphene sheets in the CSSG nanomaterials is to improve the surface area and modulate the pore size, which can be measured by the nitrogen isotherm adsorption–desorption curve (Fig. 4e) [40]. The surface area, pore volume, and average pore size can be determined by the Brunauer–Emmett–Teller (BET) analysis. As shown in Table 2, most of the CSSG nanomaterials have a larger surface area than the pristine semiconductors. For example, the surface area of MnFe_2_O_3_/GO NPs is 70.7 m^2^ g^−1^, which increases distinctly more than 8 times of the area of MnFe_2_O_3_ NPs (7.7 m^2^ g^−1^) [88]. Such high value may be due to the fact that the core–shell morphology raises the dispersion of the particles and reduces the stacking of GO nanosheets, which is a prerequisite for high electrochemical performance owing to the enhanced accessible area for the deposition of dye molecules and the exchange of the ions at the electrode–electrolyte interface. However, some CSSG nanomaterials with declining surface area are also observed, such as CdS/G NPs [80], TiO_2_/G NPs [79], and SrFeO_3_/boron-doped rGO (B-rGO) nanospheres (NSs) [89]. This may originate from the aggregation of the particles during heat treatments, the slight increase in the particle size by the shell layer, and the obstruction of the particle pores by graphene formation. In view of other properties and functions, the graphene shell should be in an appropriate amount. The content can be determined by thermogravimetric analysis (Fig. 4f) [40]. The weight percent of carbon loss may begin from the evaporation temperature of water at 100 °C to the combustion of graphene at 740 °C.

**Table 2:** Surface area S, pore volume V, and average pore size *d* of the pristine semiconductors and CSSG nanomaterials as achieved from BET analysis

Specimen	S (m^2^ g^−1^)	V (cm^3^ g^−1^)	*d* (nm)	References
a-Fe_2_O_3_/G NPs	79.5	–	20	[40]
ZnO core–shell hollow MSs	29.4	0.19	26.9	[65]
ZnO/G solid MSs	30.7	0.20	27.2	
ZnO/G core–shell hollow MSs	40.5	0.15	15.3	
ZnO/G hollow MSs	37.6	0.12	12.2	
TiO_2_(B) NWs	47.2	–	–	[51]
TiO_2_(B)/G NWs	51.1	–	1–4	
Cu_2_O MSs	29.5	–	3.4–4.1	[90]
Cu_2_O/rGO MSs	53.6	–	3.4–4.1	
CdS NPs	19	–	32.2	[80]
CdS_(-0.05)_/G NPs	9	–	35.9	
CdS_(-0.1)_/G NPs	10	–	30.2	
CdS_(-0.15)_/G NPs	10	–	39.5	
CdS_(-0.2)_/G NPs	9	–	40.0	
TiO_2_ (P25) NPs	50	–	38	[79]
TiO_2_ (rutile) NPs	24	–	38	
TiO_2_/G-5 NPs	12	–	166	
TiO_2_/G-10 NPs	9.8	–	150	
TiO_2_/G-30 NPs	7.3	–	165	
TiO_2_/G-45 NPs	9.6	–	190	
Ni(OH)_2_/RGO NSs	154	–	3–4	[93]
γ-Ga_2_O_3_ hollow NSs	81.84	–	3–4	[68]
γ-Ga_2_O_3_@RGO hollow NSs	152.95	–	3–4	
ZnO QDs	9.214	0.19	86.4	[45]
ZnO/G QDs	11.819	0.19	64.0	
ZnO/G_multilayer_ QDs	32.849	0.23	28.0	
MnFe_2_O_3_ NPs	7.7	–	–	[88]
MnFe_2_O_3_/GO NPs	70.7	–	30	
SrFeO_3_ MSs	204.76	–	0.29	[89]
SrFeO_3_/B-rGO MSs	200.404	–	0.16	

### Optical Properties

**Fig. 5:**
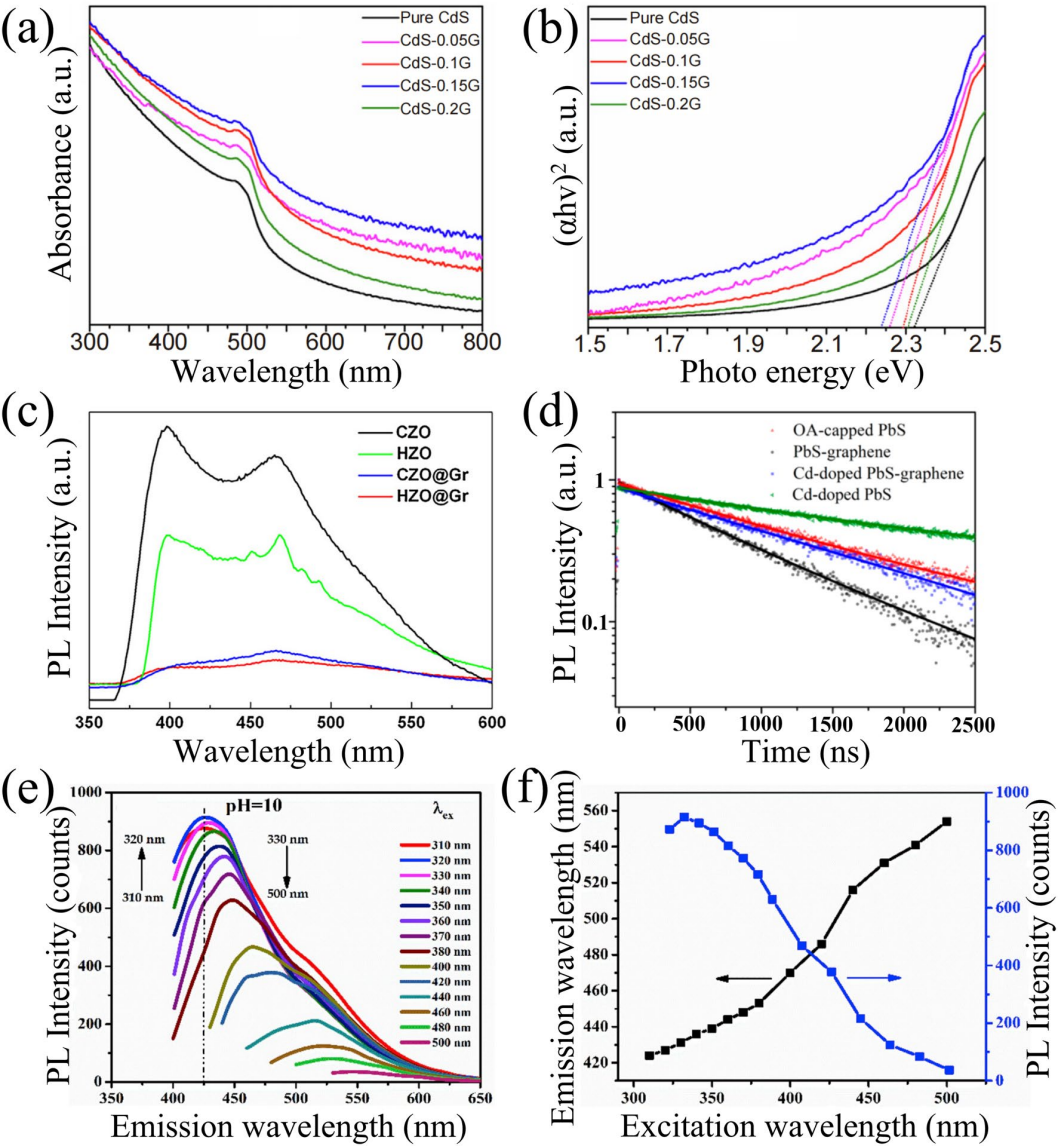
Absorption and PL spectra of the CSSG materials: **a** UV–Vis absorption spectra and **b** optical bandgap calculations of pure CdS and C-doped CdS@G samples (reproduced with permission from Ref. [80]. Copyright 2020 KeAi Publishing). **c** PL emission of commercial ZnO (CZO), hexagonal ZnO (HZO), commercial ZnO@G (CZO@Gr), hexagonal ZnO@G (HZO@Gr) NPs (reproduced with permission from Ref. [72]. Copyright 2016 Elsevier Publishing). **d** PL decay curves of PS heterogeneous QDs (reproduced with permission from Ref. [47]. Copyright 2015 ACS Publishing). **e, f** Excitation-dependent photoluminescence spectrum of prepared G QDs (reproduced with permission from Ref. [74]. Copyright 2019 Elsevier Publishing)

The light absorption ability is a critical factor that influences the performance of the compound as a photodetector or photocatalyst. By coating graphene shell, the light harvesting capability of the CSSG nanomaterials can be improved significantly, and the band-edge absorption of the semiconductor core can be adjusted to better match the required spectral range. For example, the ZnO/G NPs show an intense and broad background absorption in the visible light region, which is much stronger than the pristine ZnO NPs [72]. The phenomenon is also observed in other CSSG nanomaterials, which may be because the shell layers create mesopores with different pore size distributions. The light coming across the sample is prevented by the shell layer. For different specimens, there is a different optimal thickness for the strongest absorption (Fig. 5a) [80].

The bandgap of the compounds, as indicated by an absorption edge in the spectra, can be determined by Tauc’s plots using the following equation [9, 111]:

1 $$\left( {\alpha h\nu } \right)^{{{1}/{\text{n}}}} = {\text{A}}\left( {h\nu - E_{{\text{g}}} } \right)$$

where α is the absorption coefficient, ℎ is the Planck constant, ν is the light frequency, A is a proportionality constant, *E*_g_ is the bandgap energy, and n represents the type of bandgap with n = 0.5 and 2 for direct and indirect bandgap semiconductors, respectively. Zubair et al. found that the pure CdS owns a bandgap energy of 2.32 eV, while the bandgap is gradually narrowed as the graphene shell increases up to CdS-0.015G sample (Fig. 5b) [80]. The reduced bandgap is ascribed to the doping of carbon in the CdS matrix and the interaction of graphene and CdS. Peter et al. observed that the bandgap of TiO_2_/G QDs is closely related to the particle size [74]. The size effect is also found in PbS/G QDs [47]. Kang et al. observed that the bandgap of TiO_2_/rGO NFs is narrower than that of TiO_2_ NFs (from 2.99 to 3.19 eV) [52]. The effect is attributed to the presence of Ti–O–C bonds, which link electrons on the surface of TiO_2_ with unpaired π-electrons, causing the valence band of TiO_2_ to shift higher. The above results indicate that the electronic energy of the semiconductor core can be modulated by the graphene shell, which can achieve high electron transfer rate and delay the charge recombination more effectively. Moreover, the interfacial defects of the semiconductor core that typically act as recombination centers can be reduced significantly, as observed by the steepened absorption edges in the spectra [65, 96].

Photoluminescence (PL) spectra are widely used to investigate the efficiency of charge carrier trapping and migration, and the behavior of photogenerated electron–hole pairs. The improvement of crystal structure and electronic properties of CSSG nanoarchitectures can be illustrated by the change of position, intensity and bandwidth of the PL spectra. For instance, Zhang et al. observed two emission peaks in the PL spectra of the commercial ZnO (CZO) and hexagonal ZnO (HZO) NPs (Fig. 5c) [72]. The strong peak at 398 nm is the band-edge emission resulting from the recombination of excitonic centers, while the relative weak peak at 465 nm is the bound excitons arising from the intrinsic defects such as oxygen vacancy. The two peaks are markedly depressed for the core–shell CZO/Gr and HZO/Gr NPs due to the introduction of graphene that eliminates the surface defects and accepts the photoinduced electrons. The reduced emission intensity of the CSSG nanoarchitectures relative to the pristine semiconductors is also observed in other samples [49, 50, 72, 96], presenting lower recombination and improving separation efficiencies of the electron–hole pairs. The bandwidth of the PL spectra is closely relative to the crystal quality. Lu et al. observed a narrowed PL band of the core–shell SiC/G compared to that of the pristine SiC for the reduced interfacial defects [96]. Tavakoli et al. found that the PL spectrum of PbS/G becomes broadening for the passivation of graphene that decreases the amount of midgap states of the PbS QDs, as supported by the density functional theory simulation and time-resolved PL measurements (Fig. 5d) [47]. It is known that the excitation-dependent PL behavior is usually observed in fluorescent carbon materials [74]. Except for the intrinsic band-edge emission that keeps stable at an identical wavelength position, the PL bands of the vacancy, impurity, and defect states are sensitive to the excitation light. As shown in Fig. 5e, f [74], the increasing excitation wavelength induces the continual redshift and suppression of the emission band of G QDs that act as a coating layer on the core–shell TiO_2_/G NPs.

### Electrochemical Properties

**Fig. 6:**
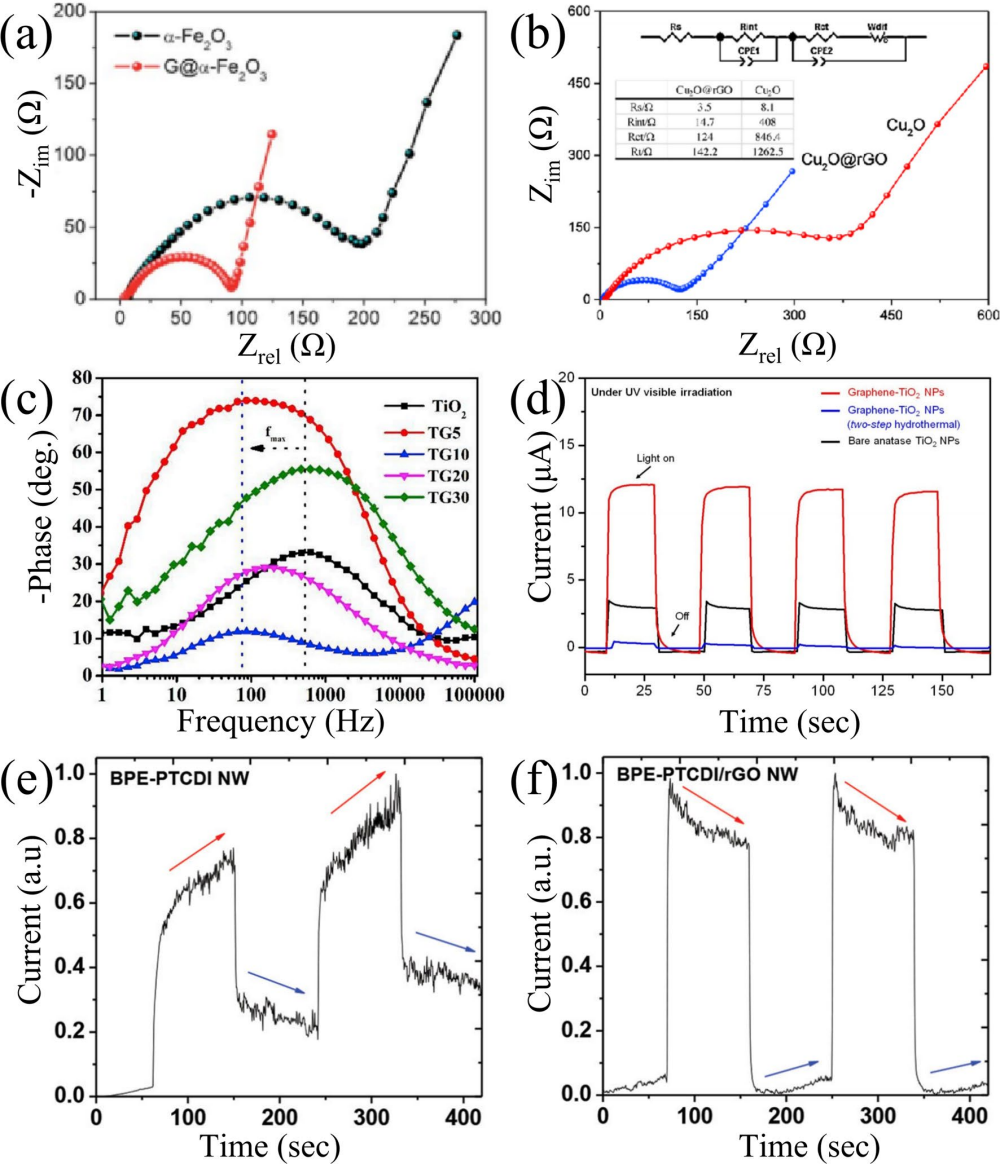
Impedance and photocurrent of the CSSG materials: **a** Nyquist plots of a-Fe_2_O_3_@G and pristine a-Fe_2_O_3_ electrode (reproduced with permission from Ref. [40]. Copyright 2011 RSC Publishing). **b** Nyquist plots of Cu_2_O@rGO and bare Cu_2_O electrode (reproduced with permission from Ref. [90]. Copyright 2015 Elsevier Publishing). **c** Bode plots of TiO_2_/G and pristine TiO_2_ QDs electrode (reproduced with permission from Ref. [74]. Copyright 2019 Elsevier Publishing). **d** Photocurrent responses of bare anatase TiO_2_ NPs, TiO_2_/G NPs (two-step hydrothermal), and TiO_2_/G NPs under UV–Vis irradiation (reproduced with permission from Ref. [21]. Copyright 2012 WILEY–VCH Publishing). Photocurrent responses of **e** BPE-PTCDI NWs and **f** BPE-PTCDI/rGO NWs (reproduced with permission from Ref. [58]. Copyright 2014 WILEY–VCH Publishing)

Electrochemical impedance spectroscopy (EIS) spectra can be used to evaluate the photoelectrochemical performance of the electrodes. The Nyquist plots of the CSSG electrodes usually exhibit a semicircle in the high-frequency region and a straight line in the low-frequency region, as typical examples in Fig. 6a, b [40, 90]. The semicircle is related to the electronic resistance and the charge transfer impedance *R*_ct_. Bigger semicircle results in a large *R*_ct_ [20]. The contact point between the semicircle at the left side and the real axis is considered as the internal resistance of the electrode material. Additionally, the linear type is mainly caused by Warburg impedance produced by the ion diffusion at the interface of the working electrode, which corresponds to the process of diffusion process. The steepest slope of the line indicates the best ion transport property. An ideal capacitor would show a vertical line. As shown in Fig. 6a [40], the semicircle diameter of α-Fe_2_O_3_/G is much smaller than that of *α*-Fe_2_O_3_, and the linear slope of the former is greater than the latter, demonstrating that the incorporation of graphene shell into the composite promotes electron transportation and ion diffusion and depresses the recombination of charge carriers in the bulk, toward ideal capacitive behavior. The similar results are also observed in VO_2_/G [87], Si/G [102], SrTiO_3_/rGO [36], Ga_2_O_3_/rGO [68], and P-Si/rGO [70]. However, in some CSSG nanomaterials, such as Cu_2_O/rGO in Fig. 6b [90], the ionic mobility changes little or even becomes worse as compared to the pristine semiconductors, though the *R*_ct_ is reduced. Zubair et al. found that the CdS/G electrode has the smallest diameter of the semicircle in light than the pure CdS electrode and all the samples in dark as well [80]. This indicates that the charge transfer resistance of the CSSG electrodes is influenced by the light irradiation.

Besides the impedance, the Bode plots as achieved in the EIS measurement allow comparing the electron lifetime, maximum phase angle, and relaxation time constant of the electrodes. Peter et al. found that the maximum frequencies (*ω*_max_) in the middle frequency region of the Bode plots for pure and graphene QDs modified TiO_2_ (TG10) are 613.13 and 75.77 Hz, respectively (Fig. 6c) [74]. Since *ω*_max_ is inversely related to the electron lifetime, the result reveals the significant increment of electron lifetime and the suppression of electron recombination of TG10. Yus et al. observed that the relaxation time constant of NiO/rGO electrode, as achieved from the Bode plots, is 4 ms lower than NiO-bare electrode (8 ms) [61], exhibiting a faster charge–discharge ability. However, the maximum phase angle declines from 76 degree of NiO-bare electrode to 70.4 degree of NiO/rGO electrode, indicating decaying capacity behavior. Moreover, the electrochemical performance of the NiO/rGO electrode can be modulated by the amount of rGO.

The photocurrent response is an effective way to detect the photoelectric conversion efficiency and separation efficiency of photoinduced electrons and holes. The intensity, rising, and falling edges of the photocurrent profile are closely relative to the band structure, charge density, impurity, and vacancy states of the working electrodes. Lee et al. found that the TiO_2_/G NPs produce much higher photocurrent under ultraviolet (UV) light irradiation, at a rate approximately 17 times larger than that of the bare anatase TiO_2_ NPs (Fig. 6d) [21], suggesting that the separation efficiency of the photoinduced electrons and holes is improved through the electronic interaction between graphene and TiO_2_. Moreover, the core–shell compound also presents strong photocurrent response under visible light (> 420 nm) irradiation, confirming high photo-activity. The photocurrent enhancement is also observed in other CSSG compounds [36, 65, 89], which may benefit from the large internal electrical field building at the interface of the semiconductors and graphene and the high mobility of carriers in the graphene. Bera et al. found that the photocurrent density of ZnO/G core–shell hollow microspheres (CSHM) is higher than that of pristine ZnO CSHM as well as other ZnO/G nanocomposites (solid MSs and hollow MSs) [65], which may originate from the superior light scattering ability, high surface area, and more active sites for electron conduction. Moreover, for pristine ZnO, the poor stability of photocurrent is found with continuous decay on prolonging the irradiation time due to the consumption of photogenerated holes as a result of self-oxidation of ZnO with existing oxygen atom on the ZnO surface, whereas the existence of graphene layers on ZnO NPs in ZnO/G CSHM offers a sufficient transport pathway of the photogenerated electrons in the system that enhances photo-stability for the direct chemical contact and charge transfer between ZnO and graphene. Shao et al. discovered that the core–shell ZnO/G NPs show a fast photoresponse under the chopped UV light, with rising and falling edges of the transient response in the time span of 9 and 11 ms, respectively [38], suggesting potential application on photodetectors. In CdS/G QDs, Zubair et al. found that the photocurrent density increases as the graphene layer thickness increases up to 4.45 mA cm^−2^ by the CdS-0.15G sample. Further increase in the graphene content results in a decrease in photocurrent density for the formation of a thicker graphene layer which hinders light absorption in CdS [80]. The result suggests an optimal thickness of the graphene shell in the CSSG nanomaterials for the high performance. Bu et al. found that the photocurrent density of ZnO/G decreases with increasing cycles of white light switching on and off [71], indicating the existence of corrosion of ZnO during the tests, which could be due to the photoelectrochemical reactions.

Instead of the stable photocurrent, a continuous decrease or increase in the photocurrent may be observed upon on-and-off switching of the light irradiation, as shown in Fig. 6e, f [58]. For the core–shell perylene diimide/rGO NWs, the declining photocurrent in light is related to the existence of depletion heterojunction layer and metastable electronic states in the bandgap, and the rapid photocurrent saturation via the rGO shell, while the enhancing photocurrent in dark is due to the synergistic interaction of the efficient exciton dissociation and more rapid charge transport via the rGO shell.

## Photocatalytic Applications

Due to the special architecture and large interfacial area, the CSSG nanomaterials can increase specific surface area, extend light absorption range, maximize light utilization, reduce electric resistance, enhance charge separation and conduction for the built-in electric field at the interfacial junction, reinforce flexibility and mechanical properties, and stabilize the structure of the core material, thus having broad applications in photocatalysis [50, 73, 96], photodetector [38], phototransistor [58], light-emitting diode (LED) [49, 98], solar cell [47, 74], lithium-ion battery (LIB) [40, 68, 91], supercapacitor [60, 71, 93], gas sensor [45, 75, 92], interfacial bonding [76, 81], filtration membrane [46], adsorber [88], and so on. The photocatalysis is widely used in energy conversion and environmental remedy. The single semiconductor materials often have low catalytic efficiency for the poor utilization of solar energy and rapid recombination of photogenerated electron–hole pairs. The emergence of CSSG nanoarchitectures provides new ideas to address these issues. In this section, the CSSG nanomaterials acting as photocatalysts will be discussed in detail, which will focus on the pollutant degradation, hydrogen generation, and carbon dioxide reduction. The parameters influencing the photocatalytic performance will be elucidated, and possible mechanisms for the enhanced photocatalytic performance will be analyzed based on the electric band alignment and charge transport of the CSSG photocatalysts.

### Degradation of Organic Pollutants

**Fig. 7:**
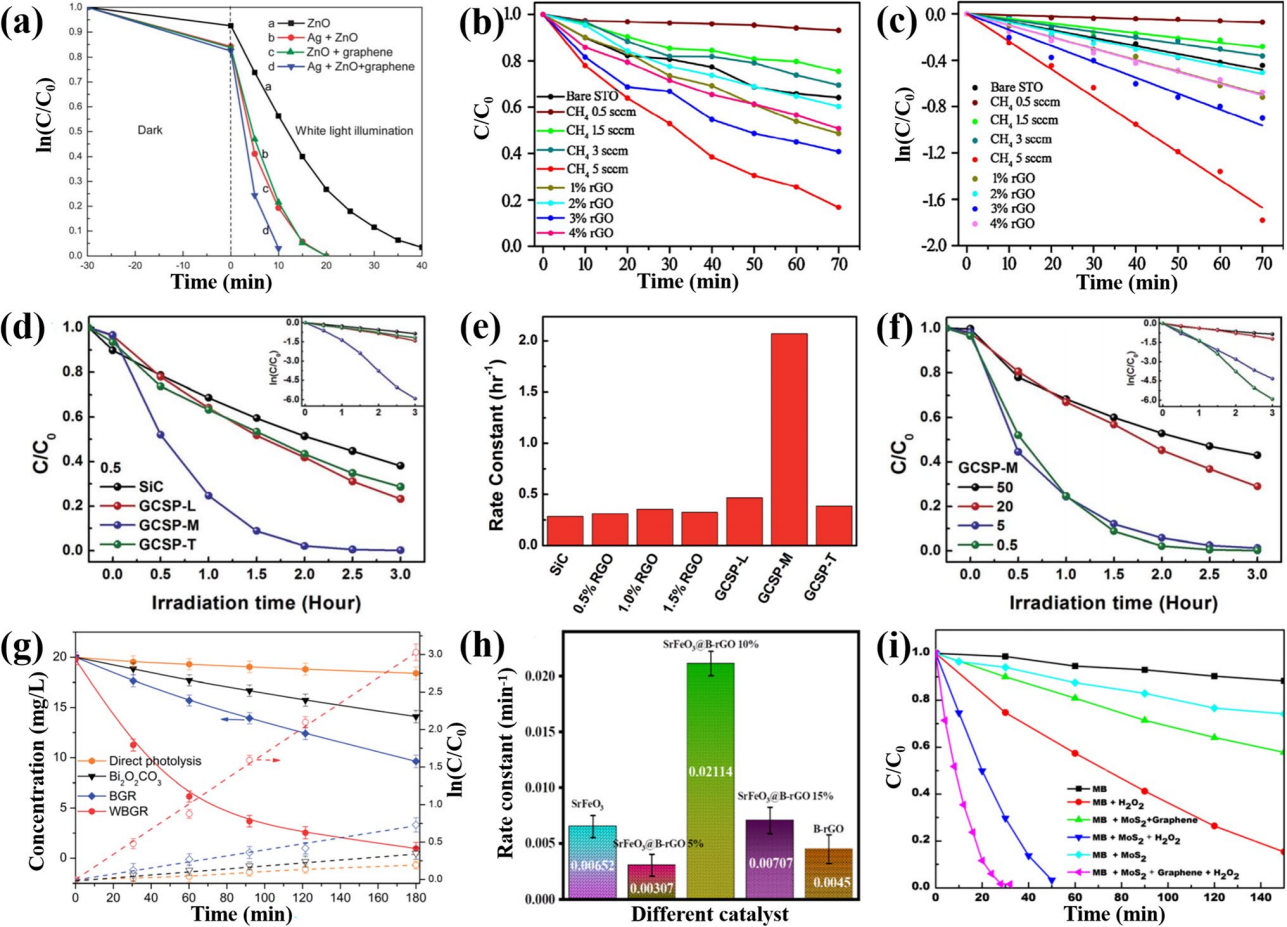
Photocatalytic activity of the CSSG materials and their pristine semiconductors: **a** Time profiles of RhB degradation in the presence of the ZnO, ZnO/Ag. ZnO/G. ZnO/G/Ag under white light illumination (reproduced with permission from Ref. [71]. Copyright 2013 RSC Publishing). **b** Time profiles of RhB degradation in the presence of SrTiO_3_ NPs and core–shell SrTiO_3_/G NPs in different growing conditions under UV light irradiation. **c** Pseudo-first-order fitting results of the RhB degradation in the presence of SrTiO_3_ NPs and core–shell SrTiO_3_/G NPs (reproduced with permission from Ref. [36]. Copyright 2017 Elsevier Publishing). **d** Time profiles of RhB photodegradation in the presence of SiC/G NPs (GCSP) covered with different graphene thicknesses together with pristine SiC powder in sizes of 0.5 mm. The inset is the natural logarithm curves corresponding to the concentration ratio, where the unit of abscissa is hour. **e** Degradation rate constants of RhB in the presence of 0.5 mm pristine SiC, corresponding rGO/SiC composites and GCSP, respectively. **f** Time profiles of RhB photodegradation in the presence of GCSP with the optimal graphene thickness in the four sizes. The inset is the natural logarithm curves corresponding to the concentration ratio, where the unit of abscissa is hour (reproduced with permission from Ref. [19]. Copyright 2014 RSC Publishing). **g** Time profiles of carbamazepine photodegradation in the presence of Bi_2_O_2_CO_3_, graphene–Bi_2_O_2_CO_3_ (BGR), and graphene-wrapped rose-like Bi_2_O_2_CO_3_ (WBGR) (solid lines) (reproduced with permission from Ref. [37]. Copyright 2014 RSC Publishing), and the apparent pseudo-first-order rate constant (dashed lines). **h** Kinetic constants of tetracycline photodegradation in the presence of SrFeO_3_, B-rGO, SrFeO_3_/B-rGO in different shell contents (reproduced with permission from Ref. [89]. Copyright 2023 ACS Publishing). **i** Time profiles of MB degradation containing different catalysts (reproduced with permission from Ref. [69]. Copyright 2017 Elsevier Publishing)

In terms of degrading organic pollutants, photocatalysis is a relatively economical and environmentally friendly method. By constructing a core–shell structure of semiconductor-graphene materials, strong oxidizing holes and reductive electrons are generated during the light irradiation, which can produce active radicals, such as hydroxyl radicals (·OH), superoxide radicals ($\bullet {\text{O}}_{2}^{-}$), and holes, for the decomposition of most organic pollutants into carbon dioxide, water, and other harmless compounds [3, 112, 113]. Bu et al. successfully removed rhodamine B (RhB) from aqueous solution using ZnO/G NPs as the catalyst [71]. The RhB dye was degraded completely in 20 min under white light (Fig. 7a). The photocatalytic activity of the specimen was significantly enhanced, with onefold improvement by the Ag modification. He et al. synthesized SrTiO_3_/G NPs as the catalyst [36]. The RhB was degraded under UV light, and the photocatalytic performance of the specimen was modulated by the shell thickness (Fig. 7b). The composite in an optimal shell thickness showed significantly enhanced photocatalytic activity compared with the SrTiO_3_, which was attributed to the special core–shell structure and chemical bond (Ti-C) for rapid interfacial electron transfer and effective electron–hole separation. The catalytic activity of the SrTiO_3_-based powders can be described by the pseudo-first-order kinetics model in Fig. 7c, which is also applied to other semiconductor materials [54, 114]. The size effect of the shell layer on the photocatalytic performance is also observed in other CSSG materials. For the SiC/G NPs, the optimal layer number of the G shell is 4–9 (Fig. 7d, e) [19]. Moreover, the core size also plays a vital role in the catalytic activity. As shown in Fig. 7f [19], the smaller size of the SiC core, the better photocatalytic performance of the SiC/G NPs, which may result from the large surface area of the small particles for the absorption and degradation of the dye molecules. Zhang et al. grew G-wrapped rose-like Bi_2_O_2_CO_3_ (WBGR) core–shell structure to maximize contact area and quantum efficiency [37]. The WBGR displayed the highest apparent rate constant (2.81 × 10^–4^ s^−1^) for carbamazepine degradation, which was 8.67 and 4.15 times higher than that of Bi_2_O_2_CO_3_ and mixed graphene-Bi_2_O_2_CO_3_ (BGR), respectively (Fig. 7g). The G shell encapsulation not only inhibited aggregation of the Bi_2_O_2_CO_3_ MSs but also protected them from structural destruction. The core–shell structures could promote photoexcited electron transfer from Bi_2_O_2_CO_3_ to G by the formation of C-Bi bonds. Preetha et al. found that the boron doping in the graphene layer can significantly improve photocatalytic performance of the core–shell SrFeO_3_/B-rGO NSs (Fig. 7h) [89], due to the enhanced electronic and transport properties while lowering resistivity. Besides the doping in the graphene layer, the ionic doping in the semiconductor cores, the introduction of vacancies, noble metal NPs and narrow bandgap semiconductors can also enhance photocatalytic activity of the CSSG materials. Furthermore, some additives in the organic solution can speed up the degradation. As shown in Fig. 7i [69], the hydrogen peroxide (H_2_O_2_) in the methylene blue (MB) solution accelerated the catalytic performance of the core–shell MoS_2_/G nanocomposite as the photoinduced electrons were immediately captured by the H_2_O_2_ to produce hydroxyl radicals.

The photocatalytic efficiency and kinetic constant of some typical CSSG nanomaterials are supplied in Table 3 for comparison [115–123]. It is found that the photocatalytic activity of the CSSG nanomaterials is modulated by the components, structure, additives, incident light wavelength, and intensity. The catalytic effect varies for different pollutants due to the different organic components and structure. The coating layer obviously enhances the photocatalytic activity. The kinetic constant of the CSSG materials with the G shell is usually larger than that with rGO or GO shell, which may result from the improved electron conduction and compact coverage of the G sheets.

**Table 3:** Comparison of photocatalytic activity of some typical CSSG nanomaterials and their pristine semiconductors for degradation of organic contaminants in aqueous solutions

Materials	Light source	Pollutant	Photocatalytic efficiency	Kinetic constant (min^−1^)	References
SrTiO_3_ NPs	Mercury 350 W, 5 mW cm^−2^	Rhodamine B	3% in 70 min	0.0068	[36]
SrTiO_3_/G NPs	Mercury 350 W, 5 mW cm^−2^	Rhodamine B	85% in 70 min	0.0239	
SrTiO_3_/rGO NPs	Mercury 350 W, 5 mW cm^−2^	Rhodamine B	55% in 70 min	0.0137	
MoS_2_ nanosheets	Xenon 500 W, distance 10 cm	Methylene blue (adding H_2_O_2_)	68% in 28 min	0.065	[69]
MoS_2_/G nanosheets	Xenon 500 W, distance 10 cm	Methylene blue (adding H_2_O_2_)	99% in 28 min	0.138	
SiC NPs	Mercury 500 W, 1 mW cm^−2^	Rhodamine B	~ 55% in 120 min	~ 0.0058	[19]
SiC/G NPs	Mercury 500 W, 1 mW cm^−2^	Rhodamine B	~ 100% in 120 min	~ 0.0333	
ZnO NPs	Xenon 300 W, distance 10 cm	Rhodamine B	100% in 40 min	–	[71]
ZnO/G NPs	Xenon 300 W, distance 10 cm	Rhodamine B	100% in 20 min	–	
ZnO/G/Ag NPs	Xenon 300 W, distance 10 cm	Rhodamine B	100% in 10 min	–	
ZnO NFs	UV lamp 24 W	Methylene blue	24% in 120 min	–	[56]
ZnO/GO NFs	UV lamp 24 W	Methylene blue	~ 97% in 120 min	–	
ZnO core–shell hollow MSs	Xenon 300 W	Rhodamine B	~ 70% in 100 min	0.012	[65]
ZnO/G hollow MSs	Xenon 300 W	Rhodamine B	~ 100% in 100 min	0.049	
ZnO/G core–shell hollow MSs	Xenon 300 W	Rhodamine B	~ 100% in 100 min	0.051	
ZnO/Co_3_O_4_ NPs	Phillips visible lamps 300 W	2,4-dichlorophenol	~ 55% in 150 min	0.017	[115]
ZnO/Co_3_O_4_/G NPs	Phillips visible lamps 300 W	2,4-dichlorophenol	91% in 150 min	0.0338	
ZnS QDs	LED 60 W	Diazinon	~ 23% in 30 min	0.019	[94]
	LED 60 W	Fenitrothion	~ 30% in 30 min	0.022	
ZnS/S-G QDs	LED 60 W	Diazinon	~ 40% in 30 min	0.029	
	LED 60 W	Fenitrothion	~ 43% in 30 min	0.031	
ZnS/S-G QDs/Ag_2_S	LED 60 W	Diazinon	> 99% in 60 min	0.053	
	LED 60 W	Fenitrothion	> 99% in 60 min	0.056	
Bi_2_O_2_CO_3_ nanoflowers	Xenon 300 W	Carbamazepine	29.6% in 180 min	0.0207	[37]
Bi_2_O_2_CO_3_/G nanoflowers	Xenon 300 W	Carbamazepine	95.4% in 180 min	0.1686	
TiO_2_ NPs	Xenon 450 W	Methylene blue	~ 10% in 60 min	0.00328	[21]
TiO_2_/G NPs	Xenon 450 W	Methylene blue	~ 90% in 60 min	0.0341	
TiO_2_ NPs	UV lamp 2 × 15 W	Rhodamine B	~ 75% in 240 min	–	[79]
TiO_2_/G NPs	UV lamp 2 × 15 W	Rhodamine B	~ 100% in 240 min	–	
TiO_2_ MSs	Xenon 500 W	Rhodamine B	~ 50% in 105 min	–	[116]
TiO_2_/GO MSs	Xenon 500 W	Rhodamine B	100% in 130 min	–	
TiO_2_ MSs	Mercury 500 W	Rhodamine B	~ 95% in 105 min	–	
TiO_2_/GO MSs	Mercury 500 W	Rhodamine B	100% in 25 min	–	
TiO_2_ NSs	Xenon 500 W	Methyl orange	~ 55% in 150 min	0.00533	[117]
TiO_2_/Au NSs	Xenon 500 W	Methyl orange	~ 58% in 150 min	0.00593	
TiO_2_/rGO NSs	Xenon 500 W	Methyl orange	~ 78% in 150 min	0.0104	
TiO_2_/Au/rGO NSs	Xenon 500 W	Methyl orange	~ 93% in 150 min	0.0168	
TiO_2_ NSs	Germicidal lamp 15 W	Methyl orange	~ 34% in 50 min	0.00837	
TiO_2_/Au NSs	Germicidal lamp 15 W	Methyl orange	~ 52% in 50 min	0.0147	
TiO_2_/rGO NSs	Germicidal lamp 15 W	Methyl orange	~ 63% in 50 min	0.0182	
TiO_2_/Au/rGO NSs	Germicidal lamp 15 W	Methyl orange	~ 91% in 50 min	0.0479	
TiO_2_ NWs	Mercury 500 W, 138.2 mW cm^−2^	Methylene blue	74.3% in 60 min	–	[50]
TiO_2_/rGO NWs	Mercury 500 W, 138.2 mW cm^−2^	Methylene blue	91.6% in 60 min	–	
rGO/TiO_2_ NSs	Metal halide 400 W cutoff filter (> 400 nm)	Rhodamine B	~ 78% in 100 min	0.01475	[118]
Au/TiO_2_ NSs	Metal halide 400 W cutoff filter (> 400 nm)	Rhodamine B	~ 85% in 100 min	0.01801	
Au/rGO/TiO_2_ NSs	Metal halide 400 W cutoff filter (> 400 nm)	Rhodamine B	~ 99% in 100 min	0.02749	
rGO/TiO_2_ NSs	Xenon 350 W	Rhodamine B	~ 90% in 100 min	0.04618	
Au/TiO_2_ NSs	Xenon 350 W	Rhodamine B	~ 90% in 100 min	0.05242	
Au/rGO/TiO_2_ NSs	Xenon 350 W	Rhodamine B	99.6% in 50 min	0.09715	
TiO_2_ hollow NSs	Mercury 500 W	Rhodamine B	80.2% in 210 min	0.0473	[67]
TiO_2_/rGO hollow NSs	Mercury 500 W	Rhodamine B	95.2% in 210 min	0.1224	
TiO_2_ hollow NSs	Xenon 500 W	Rhodamine B	54.0% in 210 min	0.0027	
TiO_2_/rGO hollow NSs	Xenon 500 W	Rhodamine B	91.8% in 210 min	0.0094	
GO/TiO_2_ MSs on electrospun polymer fibrous membrane	Xenon 500 W	Methylene blue (adding H_2_O_2_)	90.8% in 60 min	–	[46]
	Xenon 500 W	Crystal violet (adding H_2_O_2_)	92.5% in 60 min	–	
	Xenon 500 W	Methyl orange (adding H_2_O_2_)	85.4% in 60 min	–	
	Xenon 500 W	Congo red (adding H_2_O_2_)	72.0% in 60 min	–	
SnO_2_ MSs	Mercury 500 W	Methyl orange	~ 50% in 60 min	0.010	[119]
SnO_2_/rGO MSs (by chemical bonding)	Mercury 500 W	Methyl orange	~ 92% in 60 min	0.038	
SnO_2_/rGO MSs (by electrostatic interaction)	Mercury 500 W	Methyl orange	~ 73% in 60 min	0.021	
SrFeO_3_ NSs	Xenon 300 W	Tetracycline	39% in 75 min	0.0065	[89]
SrFeO_3_/B-rGO NSs	Xenon 300 W	Tetracycline	92.4% in 75 min	0.0211	
Fe_2_O_3_ NPs	Xenon 300 W	Cephalexin	40% in 60 min	–	[120]
Fe_2_O_3_@N-G NPs	Xenon 300 W	Cephalexin	90% in 60 min	–	
Fe_3_O_4_/MIL-100(Fe) MSs	Xenon 500 W	2,4-dichlorophenol (adding H_2_O_2_)	93.5% in 40 min	0.1439	[121]
Fe_3_O_4_/GO/MIL-100(Fe) MSs	Xenon 500 W	2,4-dichlorophenol (adding H_2_O_2_)	100% in 40 min	0.1969	
g-C_3_N_4_ nanosheets	Xenon 300 W, cutoff filter (< 420 nm) distance 15 cm	Doxycycline	~ 57% in 50 min	0.01632	[122]
Ag_2_CrO_4_ NPs	Xenon 300 W, cutoff filter (< 420 nm) distance 15 cm	Doxycycline	~ 62% in 50 min	0.01852	
Ag_2_CrO_4_/g-C_3_N_4_ NPs	Xenon 300 W, cutoff filter (< 420 nm) distance 15 cm	Doxycycline	~ 85% in 50 min	0.03568	
Ag_2_CrO_4_/N-G QDs/g-C_3_N_4_ NPs	Xenon 300 W, cutoff filter (< 420 nm) distance 15 cm	Doxycycline	~ 100% in 50 min	0.08839	
g-C_3_N_4_ nanosheets	Xenon 300 W, cutoff filter (> 420 nm) distance 15 cm	Doxycycline	~ 45% in 80 min	0.00705	
Ag_2_CrO_4_ NPs	Xenon 300 W, cutoff filter (> 420 nm) distance 15 cm	Doxycycline	~ 55% in 80 min	0.00962	
Ag_2_CrO_4_/g-C_3_N_4_ NPs	Xenon 300 W, cutoff filter (> 420 nm) distance 15 cm	Doxycycline	~ 88% in 80 min	0.0263	
Ag_2_CrO_4_/N-G QDs/g-C_3_N_4_ NPs	Xenon 300 W, cutoff filter (> 420 nm) distance 15 cm	Doxycycline	~ 100% in 60 min	0.04052	
BiOBr nanosheets	Neon tube, 8 mW cm^−2^	Orange II	55% in 105 min	~ 0.033	[123]
BiOBr/rGO nanosheets	Neon tube, 8 mW cm^−2^	Orange II	97% in 90 min	0.040	
BiOBr nanosheets	Hg/Xe lamp, 20 mW cm^−2^	Acetaminophen	55% in 105 min	0.003	
BiOBr/rGO nanosheets	Hg/Xe lamp, 20 mW cm^−2^	Acetaminophen	93% in 105 min	0.006	

**Fig. 8:**
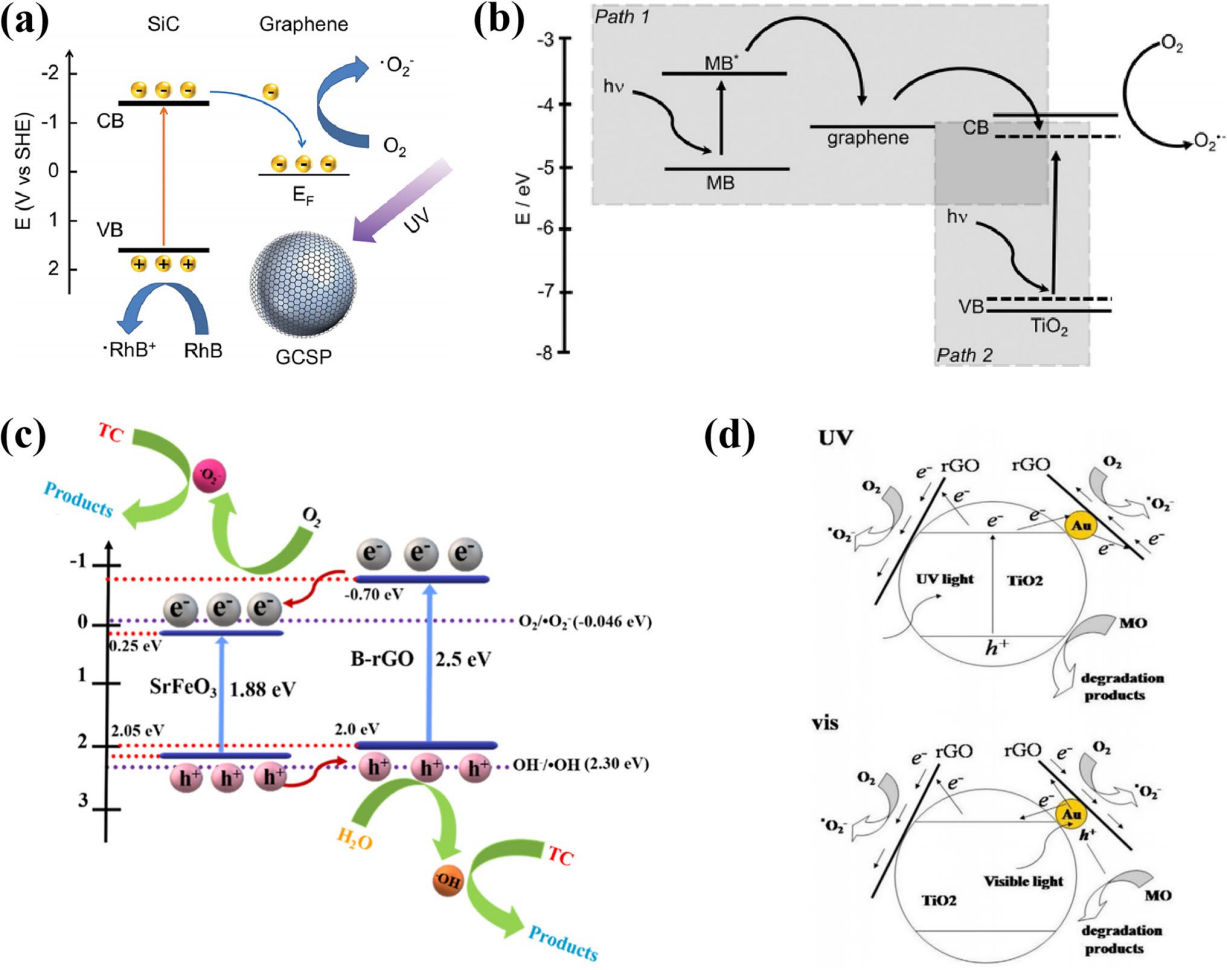
Possible enhancement mechanisms of photocatalytic performance over different CSSG catalysts: **a** SiC/G NPs (reproduced with permission from Ref. [19]. Copyright 2014 RSC Publishing). **b** TiO_2_/G NPs (reproduced with permission from Ref. [21]. Copyright 2012 WILEY–VCH Publishing). **c** SrFeO_3_/B-rGO nanocomposites (reproduced with permission from Ref. [89]. Copyright 2023 ACS Publishing). **d** TiO_2_/Au/rGO NPs (reproduced with permission from Ref. [117]. Copyright 2017 Elsevier Publishing)

Based on the band alignment of heterogeneous components and experimental characterizations, possible mechanisms for enhanced photocatalytic performance of the CSSG photocatalysts have been proposed. In the core–shell SiC/G (Fig. 8a) [19], the electrons are excited from the valence band (VB) to conduction band (CB) of SiC particles by the UV light, which transfer to the graphene shell rapidly for the high carrier transport mobility, and then are captured by oxygen in solution to produce active oxygen species. The RhB is decomposed either by the free holes in the SiC through the defect sites of graphene or by the active oxygen species. The transfer of the photoinduced electrons from the semiconductor core to the graphene shell is also proposed in MoS_2_/G [69], BiOBr/rGO [123], Fe_2_O_3_/N-G [120], and other CSSG catalysts. In the core–shell TiO_2_/G NPs, the intra-bandgap energy level of TiO_2_ is narrowed from 3.2 to 2.8 eV by direct interaction with Ti atoms and C atoms during the synthesis of graphene-TiO_2_ NPs (Fig. 8b) [21]. Under illustration of the visible light, the electrons are excited from the highest occupied molecular orbital (HOMO) level to the lowest unoccupied molecular orbital (LUMO) level of the MB molecules, which transfer to the CB of TiO_2_ by the conductive graphene layer. The valence electrons of TiO_2_ are also excited to the CB by absorbing incident light. The electrons in the CB can be trapped by oxygen molecules in the aqueous solution to form reactive oxygen species that cause the oxidative decomposition of MB molecules. The holes in the VB of the semiconductor may also participate in the pollutant degradation. This pattern resembles that in the Bi_2_O_2_CO_3_/G compound [37]. In the SrFeO_3_/B-rGO NSs, the surface potential promotes effective interaction between the pollutant and the catalyst. Additionally, the heterojunction forms a type-II band alignment, as shown in Fig. 8c [89], which facilitates the transport of the photoinduced electrons in the CB of B-rGO to the CB of SrFeO_3_, while the residual holes in the VB of SrFeO_3_ to the VB of B-rGO. The collecting electron in the CB of SrFeO_3_ will interact with O_2_ to form superoxide radicals, while the holes react with H_2_O to generate hydroxy radicals, and both of the radicals induce decomposition of the TC molecules. The enhanced charge separation and photocatalytic activity for the type-II band alignment are also observed in SnO/rGO compound [119], while in the core–shell structured TiO_2_/Au/rGO ternary photocatalyst, the promoted photocatalytic performance is attributed to the synergistic effect between Au NPs, TiO_2_ and rGO, and the existence of multi-channel electron transfer paths [117]. As shown in Fig. 8d, under UV light irradiation, the photoinduced electrons in the CB of TiO_2_ transfer to the neighboring rGO sheets directly or to the adjacent Au NPs first and then further migrate to the rGO sheets, which react with resolved oxygen nearby to generate superoxide radicals for the MO degradation, while the left holes in the VB band of TiO_2_ decompose MO. Under visible light, the electron–hole pairs in the TiO_2_ cannot be separated for low photon energy, but they can be generated in surface of the Au NPs for the surface plasmon resonance. These electrons also transport to the rGO sheets in the two paths for the MO degradation. The remaining holes in the Au NPs act as those in the VB of TiO_2_.

**Fig. 9:**
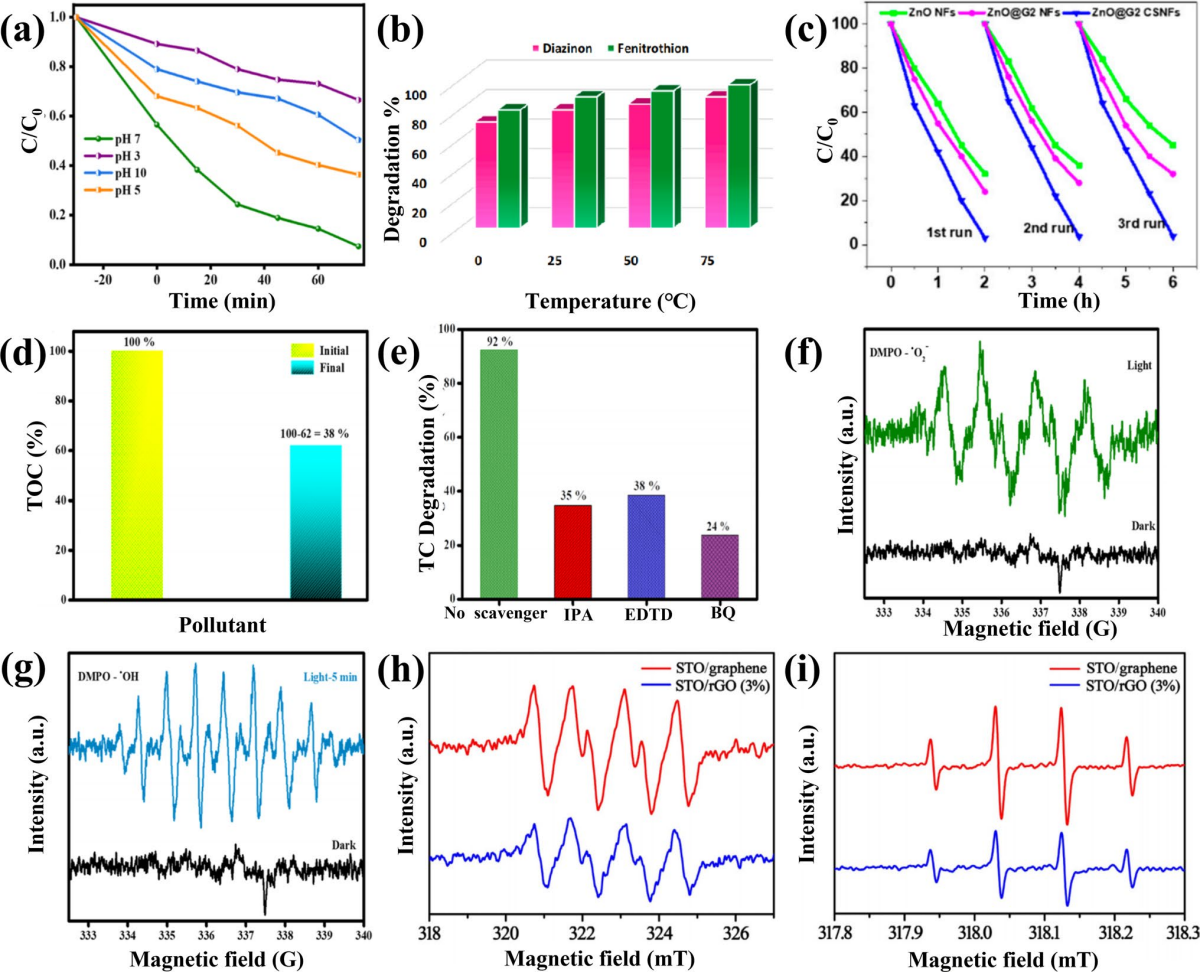
Environmental parameters, mineralization efficiency, radical trapping and detection in the photocatalytic performance of CSSG catalysts: **a** Influence of pH on the photocatalytic degradation of tetracycline in the presence of SrFeO_3_/B-rGO NSs (reproduced with permission from Ref. [89]. Copyright 2023 ACS Publishing). **b** Influence of temperature on the photocatalytic degradation of diazinon and fenitrothion in the presence of ZnS/S-G/Ag_2_S NPs (reproduced with permission from Ref. [94]. Copyright 2022 Elsevier Publishing). **c** Photocatalytic degradation cycles of ZnO NFs, ZnO NF mixed with GO (ZnO@G2 NFs), and core–shell ZnO/GO NFs (ZnO@G2 CSNFs) (reproduced with permission from Ref. [56]. Copyright 2020 MDPI Publishing). **d** TOC analysis of the degraded tetracycline in the presence of SrFeO_3_/B-rGO. **e** Scavenger test of the degraded tetracycline in the presence of SrFeO_3_/B-rGO. **f** EPR analysis of DMPO- in the presence of SrFeO_3_/B-rGO. **g** EPR analysis of DMPO-·OH in the presence of SrFeO_3_/B-rGO (reproduced with permission from Ref. [89]. Copyright 2023 ACS Publishing). **h** EPR analysis of DMPO- in the presence of SrTiO_3_/G and SrTiO_3_/rGO. **i** EPR analysis of DMPO-·OH in the presence of SrTiO_3_/G and SrTiO_3_/rGO (reproduced with permission from Ref. [36]. Copyright 2017 Elsevier Publishing)

In fact, except the catalytic properties, the photocatalytic activity of the CSSG materials is also influenced by the environment, such as pH value (Fig. 9a) and temperature (Fig. 9b) of the solution [89, 94]. Furthermore, to be a widespread photocatalyst, the stability and reusability of the catalyst are essential. The recycling experiment indicates that the photocatalytic efficiency of ZnO/GO core–shell NFs (CSNF) keeps stable in the three cycles, while that of ZnO NFs and the blend of ZnO NFs and GO powder decreases from 76 to 68% and 68% to 55%, respectively, between the first and third cycles (Fig. 9c) [56], indicating that the coating layer can eliminate or reduce the photo-corrosion effect of the ZnO core. The improved recyclability for the compacted coating layer is also observed in other CSSG catalysts. The mineralization efficiency of the organic pollutants can be detected by the total organic carbon (TOC) concentration. As shown in Fig. 9d [89], the TOC removal reaches 38% after 75 min illumination, which suggests that the core–shell SrFeO_3_/B-rGO NSs are able to degrade TC efficiently. However, the mineralization efficiency is generally lower than the degradation efficiency for the formation of certain organic intermediates. In order to verify the reactive species in the photodegradation process, the radical trapping can be conducted on the catalyst in the organic solution. Various scavengers, such as isopropyl alcohol (IPA) or tert-butyl alcohol (TBA) for the hydroxyl radicals, ethylenediamine tetraacetic acid (EDTA) or ammonium oxalate (AO) for the holes, AgNO_3_ for the electrons, and 1,4-benzoquinone (BQ) for the superoxide radicals, can be employed for the radical trapping experiment. As shown in Fig. 9e [89], the reduced efficiency in the present of IPA, EDTA, and BQ indicates that the three radicals are all involved in the degradation performance. The significant reduction in the present of BQ reveals the major role of superoxide radicals in the degradation process. The electron paramagnetic resonance (EPR) can be used to detect the radicals. The characteristic peaks of 5,5-dimethyl-1-pyrroline-N-oxide (DMPO)-$\bullet {\text{O}}_{2}^{-}$ and DMPO-·OH in Fig. 9f, g indicate that the active radicals can be only produced under light irradiation [89]. The stronger intensity of the EPR signature in Fig. 9h, i for SrTiO_3_/G than SrTiO_3_/rGO represents more active ·OH and $\bullet {\text{O}}_{2}^{-}$ radicals produced by the incident photons [36], leading to the higher photocatalytic activity.

### Generation of Hydrogen Gas

**Fig. 10:**
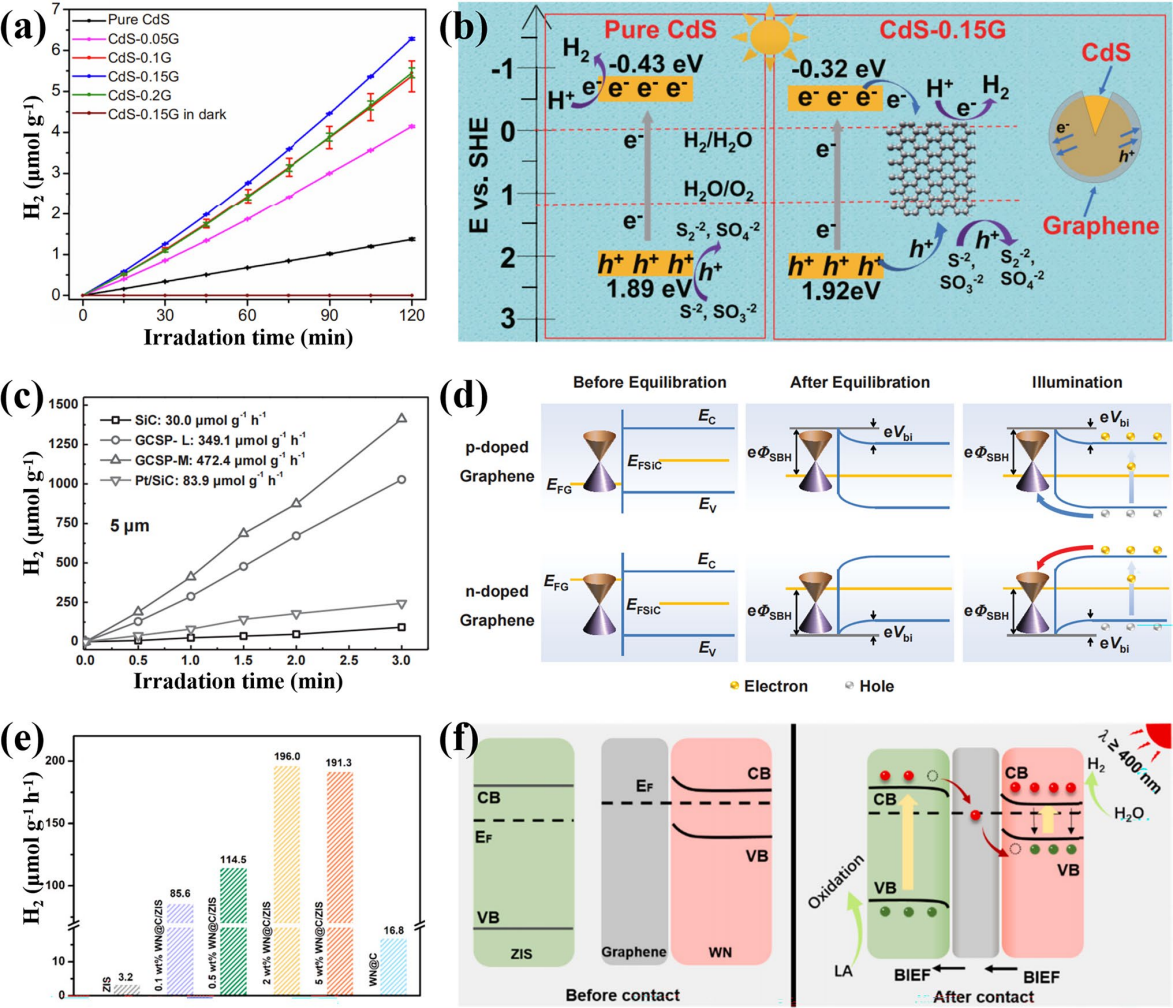
Hydrogen (H_2_) evolutions as catalyzed by different CSSG materials and their enhancement mechanisms: **a, b** CdS/G (reproduced with permission from Ref. [80]. Copyright 2020 KeAi Publishing). **c, d** SiC/G (reproduced with permission from Ref. [96]. Copyright 2015 WILEY–VCH Publishing). **e, f** WN/G/ZnIn_2_S_4_ (reproduced with permission from Ref. [124]. Copyright 2021 Elsevier Publishing)

Photocatalytic hydrogen production is one of the effective means to solve energy problems. The hydrogen gas (H_2_) can be produced from the massive water on earth. To generate H_2_ from water by the photocatalysis, the redox potentials of the catalysts should straddle the reduction potential of the H^+^/H_2_ redox pair (0 V under the normal hydrogen electrode, NHE) and the oxidation potential of the O_2_/H_2_O redox pair (1.23 V) [4, 6], which requires the bandgap of a semiconductor catalyst at least 1.23 eV and the wavelength of the incident light about 1008 nm or shorter. Some semiconductors with narrow bandgap meet the condition, but they are generally suffering rapid electron–hole pair recombination and photocorrection, while for the wide-bandgap semiconductors, the oxidation potentials are usually far more positive than 1.23 V, leading to the limited solar spectrum in the UV range. Therefore, the band engineering of the semiconductor catalysts becomes crucial for an efficient performance. Zubair et al. prepared core–shell particles of C-doped CdS and graphene for photocatalytic reduction of H_2_ [80]. The most active CdS/G (CdS-0.15G) NPs produced 3.12 mmol g^−1^ h^−1^ of H_2_ under simulated solar light (Fig. 10a), which was ~ 4.6 times superior than the pure CdS NPs, giving an apparent quantum efficiency of 11.7%. In this sample, the C doping induced the bandgap narrowing of the CdS from 2.32 to 2.24 eV, which increased the light absorption range. The photoinduced electrons in the CB of CdS transferred to graphene layer immediately for its favorable work function and then reduced H^+^ in the water to generate H_2_. The holes in the VB of CdS were also extracted by the graphene layer and neutralized by the hole scavengers in the solution to maintain the charge balance (Fig. 10b) [80]. Lu et al. prepared graphene-covered SiC particles (GCSPs) by thermal decomposition and used them as the catalyst [96]. The optimized sample achieved a high hydrogen evolution rate of 472.4 μmol g^−1^ h^−1^ in xenom light (Fig. 10c). Its photocatalytic activity exceeded the corresponding activity observed on pristine SiC particles by more than 33 times and that observed on Pt decorated SiC particles by more than 4 times, confirming superior functionality of the graphene as a cocatalyst than the noble metals. In this core–shell heterojunction, opposite charge doping (bipolar charge) regions coexisted in the graphene shell for a series of continuous facets on the SiC surface. Therefore, two inverse energy band configurations of the Schottky junction were created between the graphene and SiC, which served as the charge transfer channels for the built-in potential and the Schottky barrier (Fig. 10d) [96], leading to the efficient photocatalytic hydrogen evolution. Except for a single semiconductor component, the combination of the graphene with two or more semiconductor components in a core–shell structure may be facile and effective to extend light response, separate electron–hole pairs, and improve hydrogen generation. For instance, Gao et al. combined tungsten nitride quantum dots encapsulated in graphene (WN@C) with semiconductor ZnIn_2_S_4_ [124]. The hydrogen evolution activity of the optimal sample reached 196.0 μmol g^−1^ h^−1^, exhibiting 61 times larger than that of ZnIn_2_S_4_ and 12 times than that of WN@C under the visible light (Fig. 10e). The enhanced activity was attributed to the lower overpotential of hydrogen evolution reaction, the reduced apparent activation energy, the decreased Gibbs free energy of H adsorption, and the inhibited recombination of photo-charges for the Z-scheme band alignment of the heterojunction in Fig. 10f [124].

For comparison, the photocatalytic activities of some typical CSSG nanomaterials and their pristine semiconductors are listed in Table 4 [124–130]. It is found that the photocatalytic performance of the CSSG materials is much better than that of the pristine semiconductor. The hydrogen evolution rate of the semiconductor can be improved several to dozens of times for the combination of the conductive graphene sheets. The special core–shell structure and synergistic interaction between the core and the shell can greatly improve the electric conductivity, facilitate the mass/ion transport and gas emissions, and expose more active sites for the reactant adsorption. The structural defects and functional groups in the graphene of the CSSG materials can also increase the adsorption equilibrium thermodynamically and promote the substrate-assisted desorption pathway kinetically [131, 132] and thus lead to the enhancement of catalytic activity and long-term stability of the catalysts for overall water splitting. Secondly, the catalysts, including the single semiconductor and the CSSG material, with a narrower bandgap own a more active photocatalytic performance. For example, the bandgap of CdS (2.40 eV) is smaller than that of SiC (3.26 eV), but the hydrogen evolution rate of the former (670 µmol g^−1^ h^−1^) is much larger than that of the latter (30 µmol g^−1^ h^−1^) [69, 80]. The effect may be due to the more photoinduced charges for the extended and enhanced light excitation. However, for the hydrogen production, the redox potentials of the catalyst should keep straddling the reduction potential of the H^+^/H_2_ redox pair and the oxidation potential of the O_2_/H_2_O redox pair. Thirdly, except for the graphene, incorporating two or more semiconductor and other components in the CSSG nanomaterials can drastically improve the photocatalytic activity, which may benefit from the different light response of the different component and the effective charge separation and conduction for the built-in potential at the heterojunction.

**Table 4:** Comparison of photocatalytic activity of some typical CSSG nanomaterials and their pristine semiconductors for hydrogen generation

Materials	Light source	Sacrificial reagents	Hydrogen evolution rate (µmol g^−1^ h^−1^)	Apparent quantum efficiency (%)	References
SiC NPs	Xenon 300 W, ~ 50 mW cm^−2^	None	0	–	[69]
SiC NPs	Xenon 300 W, ~ 50 mW cm^−2^	Na_2_S/Na_2_SO_3_	30.0	–	
SiC/G NPs	Xenon 300 W, ~ 50 mW cm^−2^	None	33.2	–	
SiC/G NPs	Xenon 300 W, ~ 50 mW cm^−2^	Na_2_S/Na_2_SO_3_	472.4	5.6	
WO_3_-C_3_N_4_/G QDs	Xenon 300 W	None	16.8	–	[124]
ZnIn_2_S_4_ NPs	Xenon 300 W	None	3.2	–	
WO_3_-C_3_N_4_/G/ZnIn_2_S_4_ NPs	Xenon 300 W	None	196.0	–	
Fe_2_O_3_/rGO NPs	Xenon 250 W, cutoff filter (> 420 nm)	Triethanolamine	~ 0	–	[125]
C_3_N_4_ nanosheets	Xenon 250 W, cutoff filter (> 420 nm)	Triethanolamine	432.4	–	
C_3_N_4_/rGO nanosheets	Xenon 250 W, cutoff filter (> 420 nm)	Triethanolamine	876	–	
Fe_2_O_3_/C_3_N_4_ NPs	Xenon 250 W, cutoff filter (> 420 nm)	Triethanolamine	~ 200	–	
Fe_2_O_3_/rGO/C_3_N_4_ NPs	Xenon 250 W, cutoff filter (> 420 nm)	Triethanolamine	6607	–	
TiO_2_ NPs	Mercury 125 W and halogen 250 W	PH = 2.5	0	–	[126]
Au-TiO_2_ NPs	Mercury 125 W and halogen 250 W	PH = 2.5	~ 160	–	
Au-TiO_2_/GO NPs	Mercury 125 W and halogen 250 W	PH = 2.5	~ 456	–	
rGO/TiO_2_ NSs	Xenon 100 mW cm^−2^	Methanol	~ 95	–	[118]
Au/TiO_2_ NSs	Xenon 100 mW cm^−2^	Methanol	167	–	
Au/rGO/TiO_2_ NSs	Xenon 100 mW cm^−2^	Methanol	309	0.87	
TiO_2_/G NPs	Xenon 300 W, 150 mW cm^−2^	Methanol	52.04	~ 0	[127]
Au/TiO_2_ NSs	Xenon 300 W, 150 mW cm^−2^	Methanol	241.63	0.10	
Au/TiO_2_/G NSs	Xenon 300 W, 150 mW cm^−2^	Methanol	676.56	0.31	
TiO_2_ NPs	Xeon solar simulator 150 W, 150 mW cm^−2^	Na_2_S/Na_2_SO_3_	565	2.16	[128]
CdS NPs	Xeon solar simulator 150 W, 150 mW cm^−2^	Na_2_S/Na_2_SO_3_	673	2.57	
CdS/TiO_2_ NPs	Xeon solar simulator 150 W, 150 mW cm^−2^	Na_2_S/Na_2_SO_3_	954	3.65	
CdS/G/TiO_2_ NPs	Xeon solar simulator 150 W, 150 mW cm^−2^	Na_2_S/Na_2_SO_3_	1510	5.78	
CdS NPs	Xenon 150 W, 100 mW cm^−2^	Na_2_S/Na_2_SO_3_	670	2.7	[80]
C-doped CdS/G NPs	Xenon 150 W, 100 mW cm^−2^	Na_2_S/Na_2_SO_3_	3120	11.7	
CdS NPs	Xenon 300 W, 100 mW cm^−2^	Lactic acid	804	1.32	[63]
rGO/CdS nanosheets	Xenon 300 W, 100 mW cm^−2^	Lactic acid	2880	4.74	
CdS/MoS_2_ nanosheets	Xenon 300 W, 100 mW cm^−2^	Lactic acid	5760	9.48	
rGO/CdS/MoS_2_ nanosheets	Xenon 300 W, 100 mW cm^−2^	Lactic acid	14,400	23.7	
Cu_2_O nanocubes	Xenon 300 W, 100 mW cm^−2^	Methanol	16.8		[129]
Cu_2_O/Pd nanocubes	Xenon 300 W, 100 mW cm^−2^	Methanol	32.7		
Cu_2_O/Pd/rGO nanocubes	Xenon 300 W, 100 mW cm^−2^	Methanol	123.6		
LaFeO_3_ NPs	Xenon 300 W	Na_2_S/Na_2_SO_3_	0	–	[130]
N-G nanosheets	Xenon 300 W	Na_2_S/Na_2_SO_3_	340	–	
N-G/Pt NPs	Xenon 300 W	Na_2_S/Na_2_SO_3_	880	–	
LaFeO_3_/N-G NPs	Xenon 300 W	Na_2_S/Na_2_SO_3_	1860	–	
LaFeO_3_/N-G/Pt NPs	Xenon 300 W	Na_2_S/Na_2_SO_3_	3520	18.25	

### Reduction of Carbon Dioxide

**Fig. 11:**
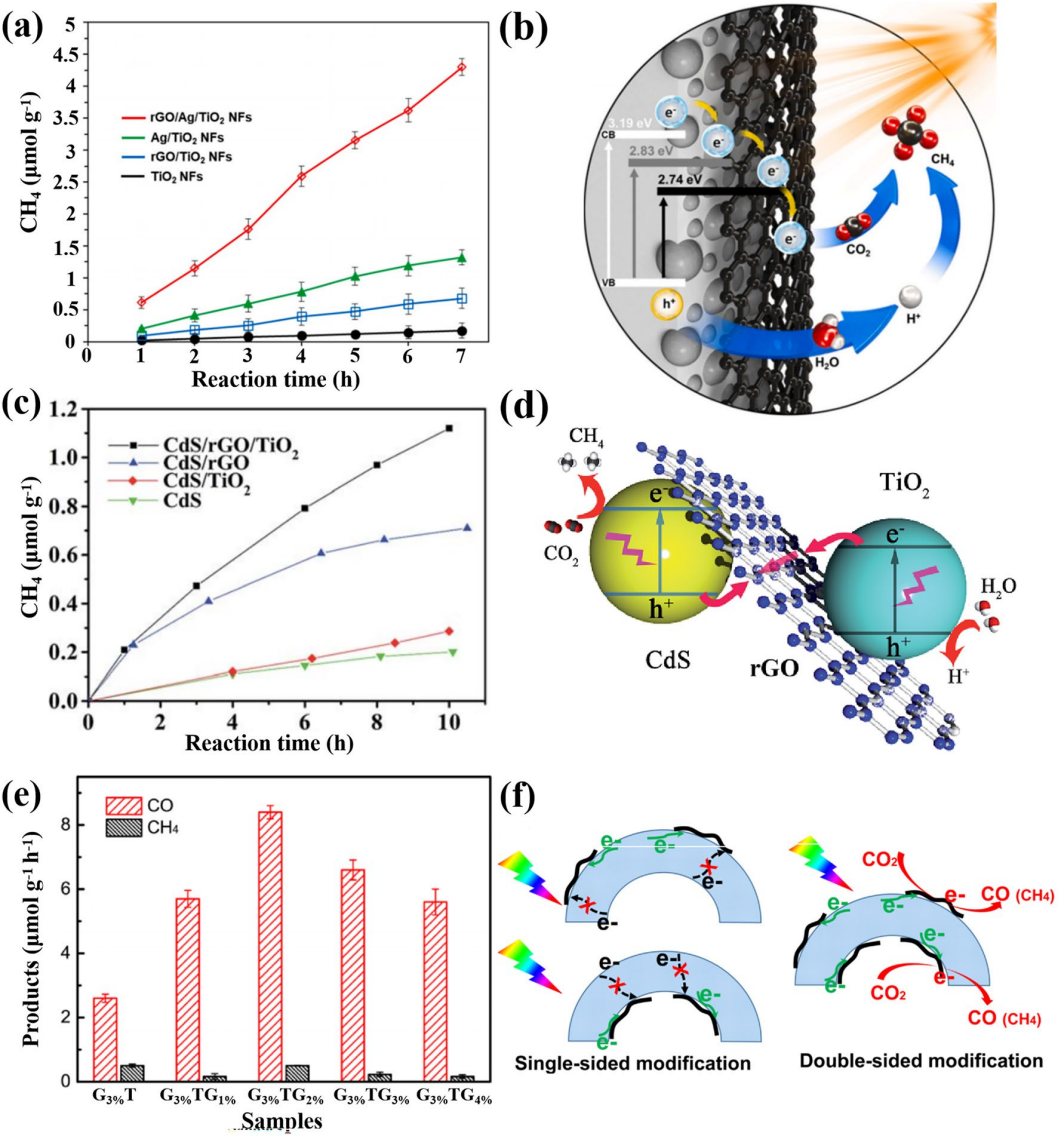
Methane (CH_4_) and carbon monoxide (CO) evolutions as catalyzed by different CSSG materials and their enhancement mechanisms: **a, b** TiO_2_/Ag/rGO (reproduced with permission from Ref. [52]. Copyright 2022 Elsevier Publishing). **c, d** CdS/rGO/TiO_2_ (reproduced with permission from Ref. [73]. Copyright 2015 RSC Publishing). **e, f** G/TiO_2_/G (reproduced with permission from Ref. [133]. Copyright 2020 Elsevier Publishing)

The global warming caused by the increasing carbon dioxide (CO_2_) in the atmosphere for social production and activities has become one of the greatest crises for human being. Converting CO_2_ into renewable fuel by photocatalytic reduction can not only reduce the concentration of CO_2_ in air but also relieve the energy shortage, which has achieved much attention as it is economically viable and environmentally friendly. Various semiconductor materials, such as TiO_2_, ZnO, CdS, Fe_2_O_3_, Cu_2_O, and WO_3_, have been developed as active photocatalysts for CO_2_ reduction in the presence of water vapor. However, the limited solar spectrum range and fast recombination of the electron–hole pairs induce low generation rate and apparent quantum efficiency (AQE), and the photogenerated holes may act as strong oxidizing agents for the corrosion of oxide semiconductor [73], leading to the poor stability and recyclability. The construction of CSSG may ideally address the issue with the graphene as a conductive and protective layer. Kang et al. fabricated rGO-wrapped Ag/TiO_2_ NFs for CO_2_ photoreduction [52]. The sample yielded 4.301 μmol g^−1^ of methane (CH_4_) in 7 h under visible light, which was 25-fold higher than the bare TiO_2_ NFs (Fig. 11a). In this ternary composite, the Ag NPs were inserted between the TiO_2_ surface and rGO sheet, which successfully prolonged the spectral reaction from UV to visible region by the LSPR effect. The Schottky barrier at the Ag-TiO_2_ junction benefited the charge separation, while the rGO layer offered a rapid pathway for trapping electrons from Ag and TiO_2_ for its outstanding electron conductivity. The transferred electrons produced an electron-enriched area on the wrapped rGO layer and converted CO_2_ to CH_4_, while the holes left on the surface of TiO_2_ reacted with H_2_O to form H^+^ and then joined the CO_2_ reduction (Fig. 11b) [52]. The NFs showed a consistent CO_2_ to CH_4_ photoreduction efficiency even after six cycles of testing for 17 h without discernible morphological change. The structural and functional stability for the rigid wrapping of the graphene shell is also observed in other CSSG catalysts, indicating unparalleled advantage of this architecture. The rGO layer as an electron mediator was also applied in the CdS/rGO/TiO_2_ core–shell nanostructure, but this time it was seated between two semiconductors [73]. The ternary compound made apparent increase of CH_4_ evolution compared to CdS, CdS/TiO_2_ and CdS/rGO (Fig. 11c). The enhanced photocatalytic performance was attributed to Z-scheme band alignment of the system that the photogenerated electrons from CB of TiO_2_ transferred to rGO, and then recombined with existing holes of CdS NSs, allowing that the photogenerated electrons enriched on the CdS semiconductor and holes on TiO_2_ for reduction of CO_2_ and oxidation of H_2_O, respectively (Fig. 11d) [73]. The improved catalytic performance by the band alignment is also observed in other CSSG materials.

Except for the band modification with various components, the enhanced photocatalytic activity of the catalysts can also be achieved by constructing CSSG materials with effective transport channel for the photoinduced charges. For example, Yang et al. fabricated TiO_2_ spherical shells with both inner and outer surface modified by graphene [133]. The optimal coating sample (G_3%_TG_2%_) displayed the highest generation rate of 8.4 µmol g^−1^ h^−1^ among the single- and double-sided modified samples, which was almost 3 times as that of G_3%_T sample (Fig. 11e). As the scheme shown in Fig. 11f [133], the graphene sheets can effectively collect and separate photogenerated electrons, but only work on those that can conduct to their surface in a short distance. The double-sided modification method greatly increases the contact area of the shell with graphene sheets, which is beneficial for graphene to separate more photoelectrons from both the inner and outer sides of the shell simultaneously.

In addition to CH_4_ gas, the CO_2_ photoreduction may produce other chemicals, such as carbon monoxide (CO), hydrogen (H_2_), oxygen (O_2_), methanol (CH_3_OH), and others. Table 5 lists a few photoreduction products as catalyzed by some typical CSSG catalysts [133–139]. The product selectivity and evolution rate chiefly rely on the adsorption/desorption properties of reactants/intermediates as well as the photocatalytic activity of catalysts [140].

**Table 5:** Comparison of photocatalytic activity of some typical CSSG nanomaterials for CO_2_ reduction

Material	Light source	Fuel	Formation rate	Apparent quantum efficiency (%)	References
CdS NSs	Xenon 300 W, distance 10 cm	CH_4_	21 µmol g^−1^ h^−1^	–	[73]
CdS/TiO_2_ NSs	Xenon 300 W, distance 10 cm	CH_4_	29.4 µmol g^−1^ h^−1^	–	
CdS/rGO NSs	Xenon 300 W, distance 10 cm	CH_4_	63 µmol g^−1^ h^−1^	–	
CdS/rGO/TiO_2_ NSs	Xenon 300 W, distance 10 cm	CH_4_	~ 117.6 µmol g^−1^ h^−1^	–	
TiO_2_ NFs	Xenon 500 W, 10 mW cm^−2^	CH_4_	~ 0.20 µmol g^−1^ in 7 h	–	[52]
TiO_2_/rGO NFs	Xenon 500 W, 10 mW cm^−2^	CH_4_	~ 0.70 µmol g^−1^ in 7 h	–	
TiO_2_/Ag NFs	Xenon 500 W, 10 mW cm^−2^	CH_4_	~ 1.30 µmol g^−1^ in 7 h	–	
TiO_2_/Ag/rGO NFs	Xenon 500 W, 10 mW cm^−2^	CH_4_	4.30 µmol g^−1^ in 7 h	–	
TiO_2_/rGO NPs	Xenon 300 W, 80 mW cm^−2^	CH_4_	6.0 µmol g^−1^ h^−1^	0.30	[134]
TiO_2_/Pt NPs	Xenon 300 W, 80 mW cm^−2^	CH_4_	13.3 µmol g^−1^ h^−1^	0.65	
TiO_2_/Pt/rGO NPs	Xenon 300 W, 80 mW cm^−2^	CH_4_	41.3 µmol g^−1^ h^−1^	1.93	
rTiO_2_/Cu-Pt/G NPs	Sunlight simulator 1000 W m^−2^	CO	394.84 µmol g^−1^ h^−1^	23.77	[135]
TiO_2_/rGO hollow MSs	Xenon 300 W	CO	3.4 µmol g^−1^ h^−1^	–	[133]
rGO/TiO_2_ hollow MSs	Xenon 300 W	CO	2.6 µmol g^−1^ h^−1^	–	
rGO/TiO_2_/rGO MSs	Xenon 300 W	CO	8.4 µmol g^−1^ h^−1^	0.034	
TaON NPs	Xenon 300 W	CH_4_	0.12 µmol g^−1^ h^−1^	0.03	[136]
TaON/G NPs	Xenon 300 W	CH_4_	1.61 µmol g^−1^ h^−1^	0.41	
CsPbBr_3_ NPs	Xenon 500 W, 100 mW cm^−2^	CH_4_/ H_2_	13.9/ 4.7 µmol g^−1^ h^−1^	–	[137]
CsPbBr_3_/GO NPs	Xenon 500 W, 100 mW cm^−2^	CH_4_/ H_2_	18.6/ 6.9 µmol g^−1^ h^−1^	–	
CsPbBr_3_/G NPs	Xenon 500 W, 100 mW cm^−2^	CH_4_/ H_2_	4.7/ 3.9 µmol g^−1^ h^−1^	–	
Fe_3_O_4_ MSs	White LED 20 W, 85 W/m^2^	CH_3_OH	278 µmol g^−1^ in 24 h	0.26	[138]
Fe_3_O_4_/CuZnO MSs	White LED 20 W, 85 W m^−2^	CH_3_OH	858 µmol g^−1^ in 24 h	0.82	
Fe_3_O_4_/CuZnO/GO MSs	White LED 20 W, 85 W m^−2^	CH_3_OH	1749 µmol g^−1^ in 24 h	1.67	
Fe_3_O_4_/CuZnO/rGO MSs	White LED 20 W, 85 W m^−2^	CH_3_OH	2656 µmol g^−1^ in 24 h	2.53	
ZnO NPs	Xenon 300 W, distance 10 cm	CO/O_2_/CH_3_OH	1.26/ 0.85/ 0.31 µmol g^−1^ h^−1^	–	[139]
ZnO/G NPs	Xenon 300 W, distance 10 cm	CO/O_2_/CH_3_OH	3.38/ 1.35/ 0.59 µmol g^−1^ h^−1^	–	

## Summary and Perspectives

In conclusion, this review highlights the CSSG nanoarchitectures for photocatalytic performance. The categories of CSSG nanomaterials along with the synthesis method, physicochemical properties, and photocatalytic performances are systematically discussed and analyzed. The CSSG nanomaterials exist in the morphologies of 0D, 1D, 2D, and 3D, which can be constructed by the internal and external driving forces. The binding effect, the amount and lattice characteristics of the graphene sheets, the photoelectric modulation of the semiconductor component, the defect states, and charge transport of the hybrid materials can be assessed by characterizing the morphology and structure, optical, and electrochemical properties of the specimens. The CSSG nanoarchitectures address key challenges of the individual semiconductors and offer opportunities for the development of more efficient and reliable photocatalysis, crucial for the future of environmental remedy and sustainable energy.

Although great achievements have been made in CSSG nanoarchitectures, several issues should be addressed in future research: (1) Uniform shell growth. To ensure the uniformity and precise control of the shell thickness and crystal structure, novel growth methods can be used for exploration. This may involve refining existing techniques, combining different construction methods, or developing new approaches to achieve consistent shell thickness across a range of core materials. (2) Core–shell modulation. It should focus on tailoring the graphene sheets, including the graphene type and layer number based on the specific physical and chemical properties of the semiconductor component. Meanwhile, the semiconductor should be regulated with an ideal composition and content. This customization can lead to CSSG nanomaterials with optimal photoelectrochemical properties for various applications. On the other hand, the graphene can be replaced by other carbon group compounds to further improve the performance of photocatalysis, such as a novel allotrope graphdiyne. Compared with graphene, graphdiyne is rich in carbon chemical bonds and tall conjugated systems, leading to strong chemical reactivity with special semiconductor characteristics [141]. The core–shell structure and synergistic interaction between graphdiyne and other semiconductors can greatly improve the electric conductivity, facilitate the mass/ion transport and gas emissions, and expose more active sites [142]. The combination can also create a S-scheme heterojunction for the effective charge separation [143] and thus enhance the catalytic activity and long-term stability. (3) Functional maximization. Except for the graphene and semiconductor, other components can also be introduced in the CSSG structures to enhance the functionality [144, 145]. For example, noble metal NPs can be encapsulated to improve the photocatalytic performance for the localized surface plasmon characteristics. The single semiconductor component can be replaced by the heterogeneous semiconductors as the latter can extend light absorption and reduce charge recombination. (4) Application extension. Besides aforementioned applications, the CSSG nanoarchitectures can also be active catalysts in other photocatalytic reactions, just like semiconductor photocatalysts in the generation of hydrogen peroxide (H_2_O_2_), organic fuels and special radicals, removal of air pollutants and hazard substances, fabrication of batteries and capacitors, inactivation of bacteria, and so on [146–151]. It is conceivable that the CSSG nanoarchitectures will perform better than single semiconductor or other kinds of semiconductor/graphene composite photocatalysts for their superior core–shell structure and physicochemical properties. Moreover, the function of the CSSG nanoarchitectures is not only limited to photocatalysis, but also has important applications in electrocatalysis, photoelectronics, supercapacitors, lithium-ion batteries, and others. (5) Mechanism illustration. Although photocatalytic mechanisms and synergistic effects of the CSSG catalysts are clear at present, the detailed functional electrocatalytic sites, wavelength-dependent charge transfer in the heterostructures, precise quantity of photogenerated electrons, reaction kinetics, doping and vacancy contributions, etc., are still elusive in most cases. Novel technologies may be used for the illustration, such as in situ irradiated X-ray photoelectron spectroscopy [152, 153] and total internal reflectance fluorescence microscopy [132]. (6) Streamlined processes. Simplifying and optimizing the synthesis processes for CSSG nanoarchitectures can lead to more efficient and cost-effective production methods, which can facilitate the scalability and industrialization of these materials. (7) Industrialization. The transition from laboratory-scale production to large-scale industrialization requires careful consideration of the scalability, cost-effectiveness, and reproducibility. Research efforts should aim to bridge the gap between small-scale research and commercial production.
